# A simple way to improve a conventional A/O-MBR for high simultaneous carbon and nutrient removal from synthetic municipal wastewater

**DOI:** 10.1371/journal.pone.0214976

**Published:** 2019-11-22

**Authors:** Dome Adoonsook, Chang Chia-Yuan, Aunnop Wongrueng, Chayakorn Pumas

**Affiliations:** 1 Department of Environmental Engineering, Faculty of Engineering, Chiang Mai University, Chiang Mai Thailand; 2 Research Program in Control of Hazardous Contaminants in Raw Water Resources for Water Scarcity Resilience, Center of Excellence on Hazardous Substance Management (HSM), Bangkok, Thailand; 3 Department of Environmental Engineering and Science, Chia Nan University of Pharmacy and Science, Tainan, Taiwan; 4 College of Resource and Environmental Engineering, Wuhan University of Science and Technology, Wuhan, China; 5 Center of Excellence in Bioresources for Agriculture, Industry and Medicine, Department of Biology, Faculty of Science, Chiang Mai University, Chiang Mai, Thailand; University of Notre Dame, UNITED STATES

## Abstract

In this study, two anoxic-oxic membrane bioreactor (A/O-MBR) systems, i.e. conventional and biofilm anoxic-oxic-membrane bioreactors (C-A/O-MBR and BF-A/O-MBR, respectively), were operated in parallel under conditions of complete sludge retention for the purposes of comparing system performance and microbial community composition. Moreover, with the microbial communities, comparisons were made between the adhesive stage and the suspended stage. High average removal of COD, NH_4_^+^-N and TN was achieved in both systems. However, TP removal efficiency was remarkably higher in BF-A/O-MBR when compared with C-A/O-MBR. TP mass balance analysis suggested that under complete sludge retention, polyurethane sponges that were added into the anoxic tank played a key role in both phosphorus release and accumulation. The qPCR analysis showed that sponge biomass could maintain a higher level of abundance of total bacteria than the suspended sludge. Meanwhile, AOB and denitrifiers were enriched in the suspended sludge but not in the sponge biomass. Results of illumina sequencing reveal that the compacted sponge in BF-A/O-MBR could promote the growth of bacteria involved in nutrient removal and reduce the amount of filamentous and bacterial growth that is related to membrane fouling in the suspended sludge.

## Introduction

The increase in nutrients, especially nitrogen and phosphorus, in domestic wastewater treatment plant discharge can cause cultural eutrophication in surface waters. The manifestations of these phenomena are known as algal blooms that occur during the warmest seasons. Excessive nutrient concentrations can accelerate the growth of microorganisms (including algae) and other aquatic plants in the receiving waters, leading to low dissolved oxygen concentrations. The improvement of nutrient removal in municipal wastewater can be achieved by biological treatment processes. The processes of biological nutrient removal, especially those involving nitrogen and phosphorus removal processes from wastewater, are widely accepted. These processes are referred to as biological nutrient removal (BNR) processes.

Since autotrophic nitrifying bacteria, namely phosphorus accumulating organisms (PAOs) and heterotrophic bacteria, compete with each other for habitat and growth under the same operational conditions, the BNR processes are not normally promising with regard to simultaneous high organic carbon and nutrient removal [1]. Therefore, it has become extremely important to develop reliable technologies that can simultaneously treat organic carbon and nutrients in municipal wastewater. Several research studies have attempted to combine the advantages of attracting growth biofilm and the membrane bioreactor (MBR) process in order to achieve some of the boundaries of traditional MBR that have been based on the activated sludge process. The application of attracting biofilm in MBR could be achieved by the addition of media (e.g., biofilm carriers) in moving or fixed bed configurations, or by the addition of aerated membranes in the bioreactor as a form of support (i.e., substratum) for biofilm growth.

The use of media in the aerobic MBR, referred to as biofilm MBR, could be a better alternative than conventional aerobic MBR because the former may increase the treatment performance by high biomass concentrations and reduce membrane fouling [2]. Several investigations have shown that phosphorus removal was successfully achieved in processes involving MBR coupled with biofilm carrier additions [3,4]. These studies have reported that the phosphorus removal was achieved by PAOs found in the anoxic/anaerobic zones in the deeper layers of the biofilm. Additionally, total nitrogen removal was reported to be higher in the biofilm aerobic MBR systems when compared to conventional aerobic MBR systems according to the authors of several published studies [2, 3]. Higher total nitrogen (TN) removal rates have mostly been attributed to simultaneous nitrification/denitrification (SND) actions that take place in deeper layers of the biofilm component where anoxic conditions have occurred. Several attached growth medias have been studied in MBR such as polyurethane sponge [3], polystyrene foam [5], polyurethane cubes [6] and polymers like MPE50 [7]. However, the polyurethane sponge has been considered a properly attached growth media that can improve organic and nutrient removal, as well as to contribute to a reduction in membrane fouling due to the fact that it can act as a bio-carrier for active biomass [4,8]. The polyurethane sponge carrier is an ideal type of attached biomass growth medium with a high porosity for microorganism immobilization, good mechanical strength, and is characterized by having a low cost [9]. It can reduce the cake layers formed on the membrane surface and retain microorganisms by incorporating a hybrid growth system for suspended and attached growths [10]. Moreover, the nitrification and denitrification rate coefficients were 1.5 and 1.6 times higher respectively in the polyurethane sponge suspended biological growth system when compared with the conventional activated sludge system [11].

A combination of anoxic and aerobic MBR compartments with sludge recirculation capabilities known as A/O-MBR has been studied frequently for carbon and nitrogen removal in wastewater treatment. Although the A/O-MBR system achieves good simultaneous carbon and nitrogen performance, the system was found to be ineffective for the treatment of phosphorus since at least three separate reactors are required to account for the conditions of differing environments (i.e., anaerobic, anoxic and aerobic), as required by the microorganisms needed in the nitrogen and phosphorus removal processes. However, the presence of an anaerobic tank in the biological nutrient removal process requires a larger footprint and is more difficult to operate than an anoxic and aerobic tank. Khan et al. [3] suggested that sponges that act as bio-carriers for attached growth media can be divided into two sub-microenvironments based on dissolved oxygen (DO) gradient values. DO concentration tends to decrease from the surface to the inside of the sponge, providing an aerobic zone at the sponge surface for heterotrophic bacteria (such as PAOs) and nitrifying bacteria. For the interior of the sponge, the anoxic/anaerobic zone is created for the purposes of denitrifying bacteria. Therefore, only two tanks (anoxic and aerobic) were involved in the process. Notably, it would be possible to achieve phosphorus release if the sponges were placed inside the anoxic compartment to create an anaerobic zone for PAOs, and the phosphate uptake would then be accomplished in the following aerobic tank. In this regard, phosphates are supposed to accumulate in greater amounts in the sludge biomass, along with dissolving in the wastewater. Consequently, the wastewater would not need further treatment via alternative processes such as ion exchange or chemical precipitation in the permeate stream.

The possibility of operating MBR without sludge withdrawal has been explored by several researchers who have focused primarily on the removal of efficiencies and other operational aspects [12,13]. All these authors have indicated the biological applicability of complete sludge retention, while reporting high and stable degradation rates and very limited sludge production. However, due to the fact that phosphorus removal is achieved by the discharge of phosphorus-enriched sludge, phosphorus removal has barely been mentioned in previous experiments with no sludge withdrawal. Consequently, popularizing MBR with complete sludge retention in practice still remains in doubt because there is an inadequate amount of knowledge on the correlation of microbial communities and nutrient removal.

The objective of this study is to introduce a simple way to achieve high simultaneous nitrogen and phosphorus removal in the A/O-MBR system by combining biofilm as active biomass that is packaged inside the anoxic compartment. The microbial community structures and compositions between the biofilm anoxic/oxic membrane bioreactor system (BF-A/O-MBR) and the conventional anoxic/oxic membrane bioreactor system (C-A/O-MBR), along with a potential link to the microbial community changes to nutrient biodegradation, were investigated to clearly understand why the biofilm that is coupled with A/O-MBR is better than the conventional A/O-MBR system in terms of biological removal by microorganisms. Moreover, within the microbial community, the differences between the adhesive stage and the suspended stage in the BF-A/O-MBR was also investigated. The microbial community that is present in the sponge biomass of the BF-A/O-MBR system and the suspended sludge of both systems was analyzed by illumina sequencing. An effort has been made to compare treatment efficiency levels and the microbial community compositions of both systems under complete sludge retention.

## Materials and methods

### Lab-scale anoxic and aerobic membrane bioreactor

**Fig 1:**
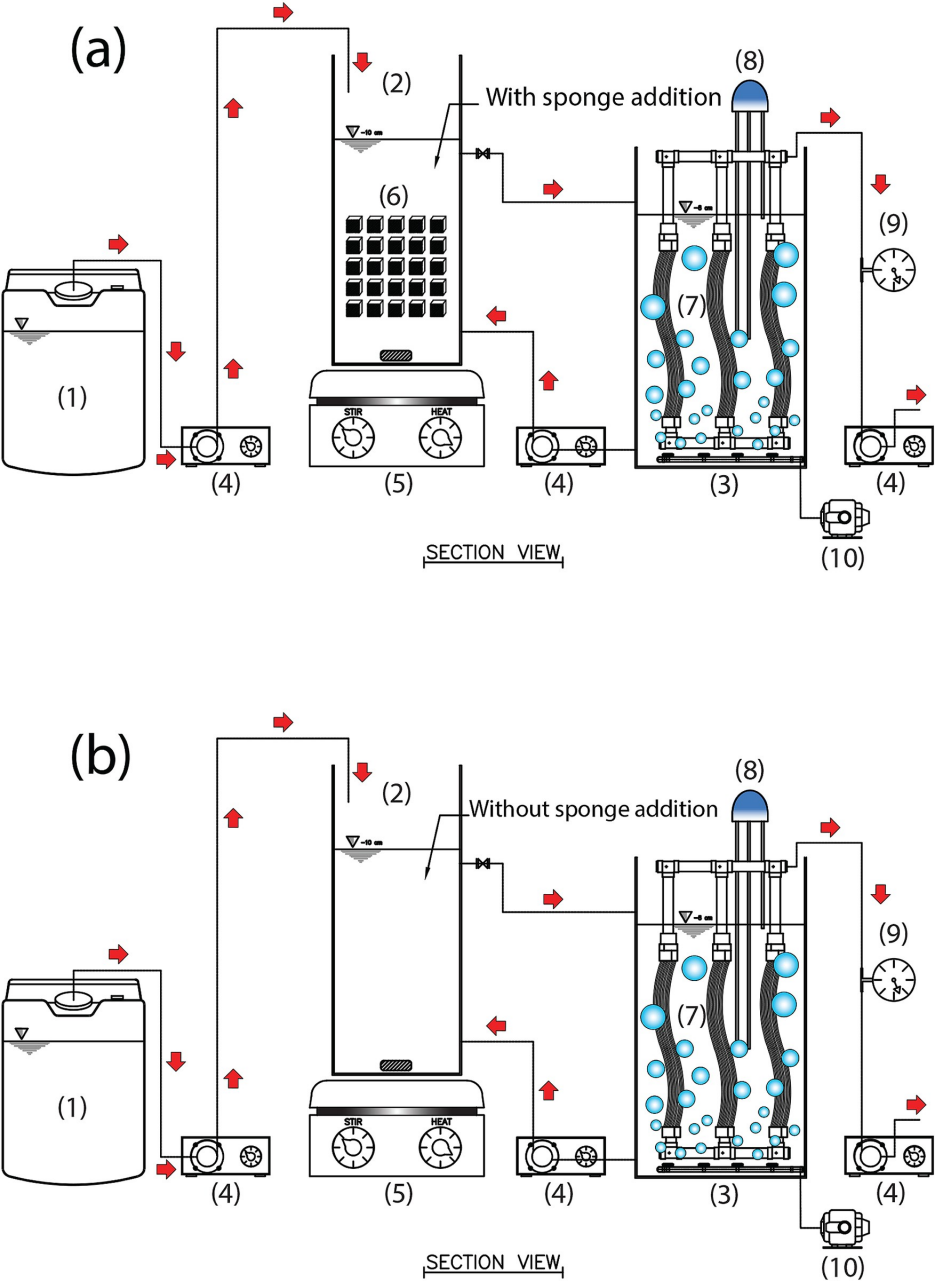
Schematic diagram of the laboratory-scale A/O-MBR system. System (a) (with compacted sponge addition) and system (b) (without compacted sponge addition) consisted: (1) feed tank; (2) anoxic tank; (3) MBR tank; (4) pump; (5) stirrer; (6) sponges; (7) membrane module; (8) level sensor; (9) pressure gauge; (10) air pump.

This study was carried out using two lab-scale anoxic and aerobic membrane bioreactors (BF-A/O-MBR and C-A/O-MBR). The reactors were operated in parallel and fed with synthetic domestic wastewater (Fig 1). Each of the two A/O-MBR systems used in this experiment consisted of two reactors with a total operating volume of 4.5 l (1.5 l for the anoxic tank and 3.0 l for the aerobic MBR tank). In the aerobic MBR tank of each system, a polyvinylidene fluoride (PVDF) hollow-fiber membrane module with a pore size of 0.1 μm and a total membrane area of 0.025 m^2^ was installed. Air diffusers were constructed beneath the membrane modules to continuously supply oxygen for biomass growth and to constantly scour the membrane surface for potential fouling control. The dissolved oxygen (DO) concentrations in the anoxic tank and the aerobic MBR tank were controlled at approximately 0.3 mg/l and 2.0 mg/L, respectively. A pressure gauge was installed to measure the transmembrane pressure (TMP) during the filtration period.

As for the BF-A/O-MBR system, 50 pieces of one cubic centimeter polyurethane sponge with a density of 30 kg/m^3^ were compacted inside the anoxic tank with a 10% volume fraction of the anoxic tank. In both systems, synthetic wastewater from the feed tank was introduced to the anoxic tank using a peristaltic pump (Model no. 7553–71, Masterflex). The anoxic effluent was made to flow to the aerobic MBR tank using gravity. A water level controller was installed in the aerobic MBR tank of each system to maintain a constant water level corresponding to a total hydraulic retention time (HRT) of 9 h (3 h for anoxic tank and 6 h for aerobic MBR tank). A permeate flux of 20 L/m^2^.h with a permeate flow rate of 14.4 l/day was maintained during the entire experimental procedure. Intermittent permeation with 5 min suction and 1 min pause was applied to minimize membrane fouling. The system was operated at ambient temperatures. The suspended sludge in the aerobic MBR tank was recirculated to the anoxic tank using a return pump with a recirculation rate of 2 times the influent flow rate. It is important to note that all of the systems were operated without sludge withdrawal except when it was necessary to analyze certain relevant parameters.

The raw wastewater employed in this study was synthetic domestic wastewater imitated from domestic wastewater characteristics in Taiwan with concentrations of 210 ± 5 mg/L chemical oxygen demand (COD), 60 ± 1 mg/L total nitrogen (TN), and 5.5 mg/L total phosphorus (TP). The components of the synthetic wastewater were as follows: C_2_H_5_COONa (200 mg/l), NH_4_Cl (200 mg/l), KH_2_PO_4_ (28 mg/l), NaHCO_3_ (220 mg/l), MnCl_2_.4H_2_O (0.19 mg/l), ZnCl_2_.2H_2_O (0.0018 mg/l), CuCl_2_.2H_2_O (0.022 mg/l), MgSO_4_.7H_2_O (5.6 mg/l), FeCl_3_.6H_2_O (0.88 mg/l), and CaCl_2_.2H_2_O (1.3 mg/l). The reactors were inoculated with activated sludge from a domestic wastewater treatment plant located at An-Ping Tainan, Taiwan. A sample of the activated sludge collected from the aeration basin was settled for 4 h, and the supernatant was discarded. At the start of each system, a portion of activated sludge was diluted with DI water to 10 liters, aerated in the container and fed with a stock solution at an influent flow rate of 3.5 liters/day to restore its activity. After 7 days, a 4.5-liter volume of the renewed sludge was applied as an inoculum to maintain an initial MLSS concentration of approximately 5,000 mg/l.

### Analytical method for wastewater quality parameters

Mixed liquor suspended solids (MLSSs), mixed liquor volatile suspended solids (MLVSSs), chemical oxygen demand (COD), ammonia nitrogen (NH_4_^+^-N), nitrite (NO_2_^−^N), nitrate (NO_3_^−^N), total nitrogen (TN), and total phosphorus (TP) concentrations were measured according to the standard methods [14]. Dissolved oxygen and pH were determined using a WTW Multi pH/Oxi 340i dissolved oxygen meter. A differential pressure gauge was used to measure the transmembrane pressure (TMP) of the membrane module. As for the sponge biomass calculation, certain amounts of dried one-cubic-centimeter-sponge samples without attached growth were weighed before being submerged in the anoxic tank. Sponge samples with attached growth were then taken from the anoxic tank. The sponge samples were dried, and the mass of the sponges was weighed. Afterwards, the mass of the sludge in the sponges was measured by comparing the mass of the sponge samples before and after being submerged in the anoxic tank. Finally, the sponge biomass in the samples was calculated as follows.

$$Sponge\;biomass\;(mg/l)=\frac{[Mass\;of\;sludge\;in\;sponge\;(g)-Mass\;of\;clean\;sponge\;(g)]\times 10^{6}}{Sponge\;volume\;(cm^{3})}$$

Samples of mixed liquor for extracellular polymeric substance (EPS) analysis were taken from the anoxic tank and the aerobic MBR tank. All samples were cooled to 4°C to mitigate microbial activity. The samples were then centrifuged at 6,000 g for 20 min at a temperature of 4°C. The supernatant was filtrated with a 0.45 μm membrane filter. The precipitation was re-suspended in the buffer solution with the same volume of the supernatant. The extraction of EPS was based on the cation exchange resin (Dowex^®^ Marathon^®^ C, Na^+^ form, Sigma-Aldrich, Bellefonte, PA) extraction method, as has been described by Frølund et al. [15]. The exchange resin of 70 g of Dowez/g MLVSS was added to 30 ml of the sample. The sample and the exchange resin were then mixed at 600 rpm (4°C) using a magnetic stirrer for 2 h. After that, the samples were centrifuged for 15 min at 12,000 g in order to remove the suspended solids. The supernatant was then filtered through a 0.22 μm membrane filter. The filtrate was presented as the total EPS. The carbohydrate content and the protein content were measured using the phenol-sulfuric acid method and a Modified BSA kit based on the methods described by Dubois et al. [16] and Lowry et al. [17], respectively. Glucose and bovine serum albumin (BSA) were used as the carbohydrate standard and the protein standard, respectively. The sum of the carbohydrate and the protein EPS concentrations was presented as the total EPS concentration value.

### Phosphorus mass balance analysis

**Fig 2:**
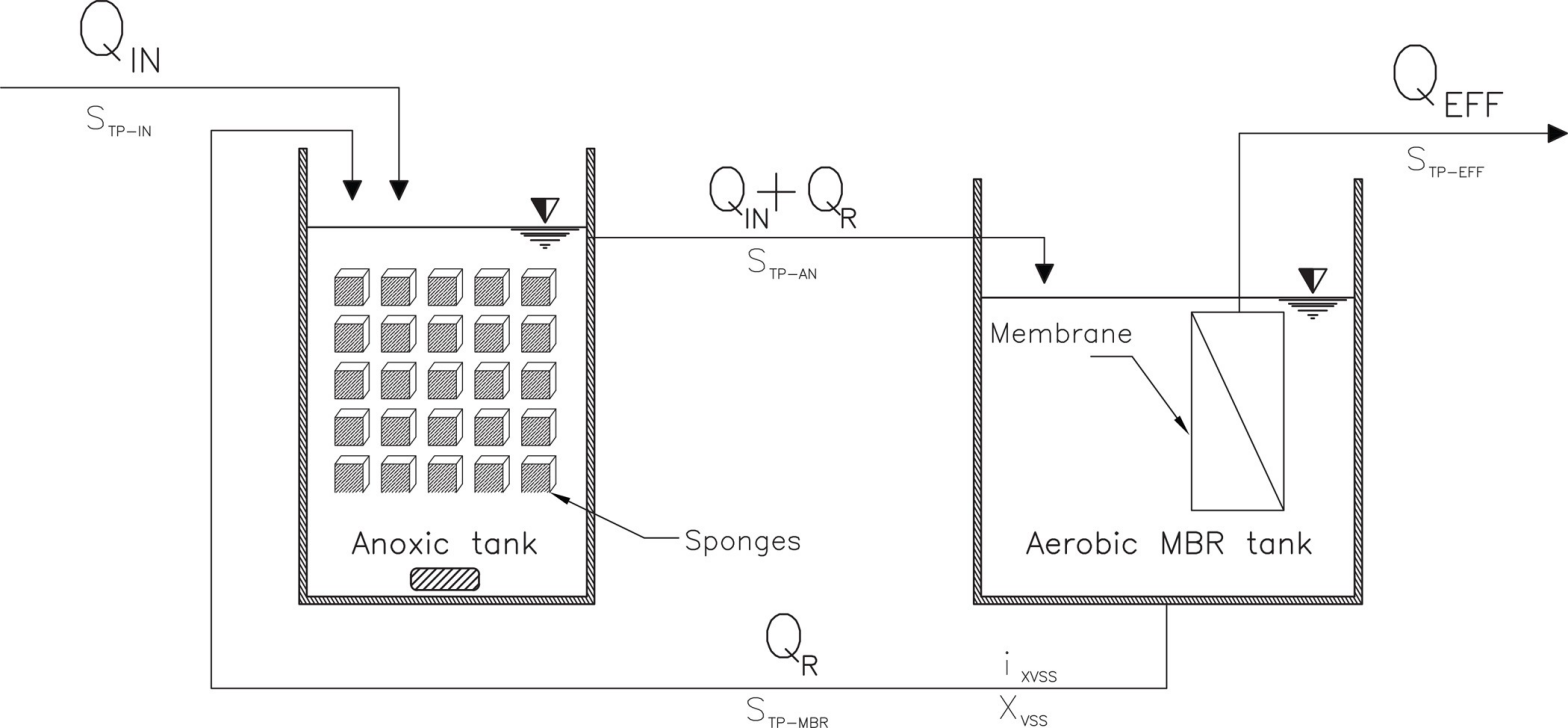
Schematic diagram of A/O-MBR used in this study (S = concentration of TP in different zones; Q_IN_ = influent flow; Q_EFF_ = effluent flow; Q_R_ = sludge returning flow; i_XVSS_ = nitrogen or phosphorus content by weight of MLVSS concentration (%); XVSS = MLVSS concentration).

The phosphorus mass balance was calculated according to Fig 2. The phosphorus in the whole system was considered as phosphorus release and uptake. The total mass of phosphorus in the influent is the sum of the mass of PO_4_^3—^P in the influent flow.

$$Feeding\;P=Q_{IN}\times S_{TP-IN}$$

where; *S*_*TP-IN*_ is influent TP concentration (mg/l).

The phosphorus mass balance in the anoxic tank can be expressed as

$$Q_{IN}\times S_{TP-IN}+Q_{R}\times S_{TP-MBR}=\left(Q_{IN}+Q_{R}\right)\times S_{TP-AN}+\Delta P_{release}$$

The phosphorus mass balance in the aerobic tank can be expressed as

$$\left(Q_{IN}+Q_{R}\right)\times S_{TP-AN}=Q_{EFF}\times S_{TP-EFF}+[Q_{R}\times i{\left(TP\right)}_{XVSS}\times X_{VSS}]+\Delta P_{uptake}$$

where; *S*_*TP-MBR*_ is TP concentration of MBR tank (mg/l), *S*_*TP-AN*_ is TP concentration of anoxic tank (mg/l), *S*_*TP-EFF*_ is TP concentration of effluent (mg/l), *ΔP*_*release*_ is the mass of phosphorus release (g/day), *ΔP*_*uptake*_ is the mass of phosphorus uptake (g/day) and *i(TP)*_*XVSS*_ is TP content by weight of MLVSS concentration (%).

### Sampling and DNA extraction

The sponge biomass of the BF-A/O-MBR system and the sludge biomass in the aerobic MBR tank of both systems were sampled periodically throughout a 45-day period. DNA extraction was performed using the Fast-DNA SPIN kit for soil (MP Biomedicals, Solon, OH, USA). The final DNA concentration and purification were determined by NanoDrop 2000 UV-vis spectrophotometer (Thermo Scientific, Wilmington, USA). Nucleic acid measurement was chosen as the mode to measure DNA. Nucleic acid measurement with this equipment was calculated using Beer-Lambetr equation modification according to the equation shown below. The average DNA concentrations of each sample are shown in S1 Table. The extracted DNA was then evaluated on 1% (wt/vol) agarose gel and stored at -20°C for further use.

c=(A×ε)/b

where; c = the nucleic acid concentration (ng/μL), A = the absorbance in AU, ε = the wavelength-dependent extinction coefficient in ng-cm/μL, b = the pathlength in cm

### Quantitative real-time PCR assays

For each sample, a quantitative real-time PCR was carried out in duplicate with a LC 480 SYBR Green QPCR Master Mix (Roche Diagnostics, Mannheim, Germany) in a light Cycler 2.0 system instrument. The quantification of bacterial AOB *amoA* genes, denitrifying *NirS* genes, and total bacterial 16S rRNA genes was performed using the primers (S2 Table). The 10-fold dilution series of standard calibration ranged from 5.0 to 5x10^7^ copies/reaction. The PCR mixture at a volume of 20 μl contained the reagent shown in S3 Table. The DNA template was changed to ddH_2_O when conducting the negative control. The PCR mixture was the same for all genes.

### Bacterial community analysis using illumina sequencing

The sponge biomass samples of the BF-A/O-MBR system and the sludge biomass samples of both systems on day 45 were chosen for illumina sequencing analysis. PCR amplification was carried out immediately after DNA extraction using primers targeting the V3 and V4 regions of the 16S rRNA gene with the forward primer 341F (5’- CCTACGGGAGGCAGCAG-3’) and the reverse primer 805R (5’-GACTACCAGGGTATCTAATCC-3’), all of which used the extracted DNA as a template. The PCR mixture was composed of 0.8 μl for each forward and reverse primer (10 μM, Metabion, Germany), 3 μl of the template DNA for the samples, and 12.5 μl of 1x of Hot Master Mix (Promega GoTaq® Green Master Mix) to a final volume of 25 μl. For the negative control, 3 μl of the elution solution was used. The amplifications were performed under the following conditions: initial denaturation at 95°C for 2 minutes, followed by 30 cycles of denaturation at 95°C for 30 seconds, primer annealing at 60°C for 30 seconds, and extension at 72°C for 30 seconds with a final elongation period at 72°C for 5 minutes.

Sequencing was carried out by using paired-end Illumina MiSeq sequencing on an Illumina MiSeq device (Illumina Inc., San Diego, CA, USA) with 600 cycles (300 cycles for each paired read and 12 cycles for the barcode sequence) according to the manufacturer’s instructions. To artificially increase genetic diversity, it has become common practice to mix the specimens in a controlled library of genomic DNA from the phage phix to prevent focusing and phasing problems due to the sequencing of “low diversity” libraries. Sequence analysis was conducted using the 16S-based metagenomics workflow of MiSeq Reporter version 2.6.2.3 (Illumina). Samples were gathered into a single library for sequencing on the Illumina MiSeq sequencing system which generated paired 300 bp reads. Sequences were then demultiplexed based on index sequences. FASTQ files with Quality Score Encoding were created. Operational Units (OTUs) were clustered with 97% similarity cut-off using UPARSE (version 7.1 http://drive5.com/uparse/). Chimeric sequences were identified and removed using UCHIME. The phylogenetic affiliation of each 16 S rRNA gene sequence was analyzed by RDP Classifier using a confidence threshold of 70%. OTUs clustering and classification at several taxonomic levels including kingdom, phylum, class, order, family, genus, and species, were performed. The raw sequencing data and the processed data files are available at https://www.ncbi.nlm.nih.gov/geo/query/acc.cgi?acc=GSE135797.

### Sequence analysis

The diversity of the microbial community was quantified by Shannon Weiner’s Diversity Index and Simpson’s index [18]. Simpson’s index is heavily weighted toward the most abundant species in the sample while being less sensitive to species richness. The Shannon index is positively correlated with species richness and evenness and gives more weight per individual than the common species, being sensitive to sample size [19]. Higher numbers indicate greater levels of diversity. Typically, values are generally between 1.5 and 3.5 in most ecological studies, and the index is rarely greater than 4. The ensuing formula was used to calculate the values as:

$$H^{\prime }=-\sum_{i=1}^{R}pi\;\text{ln pi}$$

where H′ = Shannon Weiner’s Diversity Index, pi = ratio of individuals in the i species, R = number of species

$$E=\frac{H'}{in\;S}$$

where E = evenness, S = total number of species in the population.

The species richness (H_max_) was calculated as:

$${\text{H}}_{\text{max}}=\text{ln S}$$

Simpson’s index (D) of the samples was calculated as:

$$D=\sum Pi^{2}$$

Bacteria assemblage patterns were examined utilizing several approaches including R Project for Statistical Computing version 3.4.2 supported by CRAN (R Core Team, 2017). Sampling runs were grouped based on microbial communities using Hierarchical Cluster Analyses (HCA) and carried out with the h cluster function of R using Euclidean distance. The complete option was embraced for clustering as it includes a greater proportion of the information than 2%. Pearson’s product momentum correlation coefficient was used to estimate the linear correlation between the abundance of the top 37 most abundant OTUs and the two most important environmental variables (nitrogen and phosphorus). Pearson’s coefficient (r_p_) was always between -1 and +1, where -1 means a perfectly negative correlation and +1 indicates a perfect positive correlation, while 0 indicates the absence of a relationship.

## Results and discussion

### MBRs performance

General performance of the two MBR systems is summarized in Table 1. The COD and NH_4_^−^-N removal performances were higher than 97% and 99%, respectively, when the influent COD and NH_4_^−^N loads were 0.65 kg-COD/m^3^.d and 0.16 kg- NH_4_^−^N /m^3^.d, respectively. Since autotrophic nitrifying bacteria are well-known as slow-growing bacteria [20], the extended mean cell residence time (MCRT) would be able to provide nitrifying bacteria that is sufficient enough to ensure effective nitrification. Therefore, the complete sludge retention applied in this study was very beneficial for the nitrifying bacteria. However, the TN removal efficiency levels were found to be significantly higher (p = 0.05) in the BF-A/O-MBR system (87.79%) than in the C-A/O-MBR system (81.48%). The results indicate that the reactor configuration (i.e., compacted sponge addition) played a role in denitrification and TN removal. The higher level of TN removal efficiency associated with the BF-A/O-MBR system can mostly be attributed to the multifunctional microbial reactions that occurred in the developing sponge biofilm [2], especially with regard to denitrifying bacteria and DNPAOs, both of which play crucial roles in nitrogen removal. This aspect will be proven using real-time PCR and illumina sequencing analysis in the next sections.

**Table 1:** Influent concentration, effluent concentration and system performance of BF-A/O-MBR system and C-A/O-MBR system.

System types	BF-A/O-MBR	C-A/O-MBR
Influent COD (mg.l^-1^)	246.65 ± 9.0	242.06 ± 8.6
Effluent COD (mg.l^-1^)	7.44 ± 2.9	4.07 ± 2.0
COD removal efficiency (%)	96.98 ± 1.2	98.32 ± 0.8
Influent NH_4_^+^ (mg.l^-1^)	59.93 ± 7.4	56.76 ± 4.4
Effluent NH_4_^+^ (mg.l^-1^)	0.38 ± 0.1	0.57 ± 0.02
NH_4_^+^ removal efficiency (%)	99.39 ± 0.2	98.92 ± 0.1
Influent TN (mg.l^-1^)	60.14 ± 7.4	58.38 ± 5.0
Effluent TN (mg.l^-1^)	7.34 ± 0.7	10.81 ± 1.0
TN removal efficiency (%)	87.79 ± 2.0	81.48 ± 2.6
Influent TP (mg.l^-1^)	5.05 ± 0.2	5.12 ± 0.2
Effluent TP (mg.l^-1^)	0.24 ± 0.6	3.89 ± 0.2
TP removal efficiency (%)	95.30 ± 1.2	24.02 ± 5.3
MLSS (mg/l)	13142 ± 119	9032 ± 69
MLVSS (mg/l)	10910 ± 201	8530 ± 136
EPS protein (mg/gMLVSS)	77.03 ± 14.6	41.32 ± 5.6
EPS carbohydrate (mg/gMLVSS)	51.10 ± 26.4	67.45 ± 13.8
Total EPS (mg/gMLVSS)	128.13 ± 9.1	108.77 ± 5.0

Surprisingly, the extremely high phosphorus removal performance was observed in the BF-A/O-MBR system with an average removal efficiency of 95.30%, while the average phosphorus removal performance was lower than 25% in the C-A/O-MBR system. Normally, phosphorus present in wastewater streams can be divided into a soluble form (e.g., orthophosphate) and an insoluble form (e.g., organic and polyphosphate). The soluble form of phosphate can easily pass through ultrafiltration membrane pores, while insoluble forms of phosphate cannot. The biological phosphorous removal efficiency in the MBR systems is generally dependent upon the soluble phosphorus that is present in the wastewater stream, as it becomes insoluble in the sludge waste of the PAOs activities (luxury uptake), while the efficiency of the solid separation process is influenced by the membrane. Phosphorus removal is achieved by the discharge of phosphorus-enriched sludge. However, the activated sludge reached the maximum capacity to store phosphorus when SRT was over 40 d, and was not able to uptake more phosphorus from the mixed liquor to ensure that the effluent TP concentration was down to 0.5 mg/L [21]. Therefore, phosphorus removal has rarely been mentioned in the previous experiments that revealed no sludge discharge. Because both systems operated without sludge withdrawal (except in the analysis), the very high phosphorus removal performance in the BF-A/O-MBR system was unexpected.

The TP mass balance showed that the average TP feeding rates were 0.061 and 0.062 g-P/day, and that the average TP releasing rates of around 1.31 and 0.03 g-P/day occurred in the anoxic tanks of the BF-A/O-MBR and C-A/O-MBR systems, respectively (S4 Table). The uptake rates in the aerobic MBR tanks in the BF-A/O-MBR and C-A/O-MBR systems were found to be 2.41 and 0.18 g-P/day, respectively. The results indicate that when the sponge biomass was present in the anoxic tank, and consequently, it could create anaerobic conditions inside the sponge biomass for the release of phosphates by PAOs. Khan et al. [3] suggested that sponges that act as bio-carriers attached growth media could be divided into two sub-micro-environments based on the dissolved oxygen (DO) gradient. DO concentration tends to decrease from the surface to the inside of the sponge, providing an aerobic zone at the sponge’s surface for heterotrophic bacteria (such as PAOs) and nitrifying bacteria. For the interior of the sponge, the anoxic/anaerobic zone was created for the purposes of denitrifying bacteria and PAOs. The mass balance result also showed that most of the TP in the influent was adsorbed somehow into the suspended biomass at 0.015 g-P/day and the sponge biomass at 0.044 g-P/day in the BF-A/O-MBR system. Meanwhile, in the C-A/O-MBR system, 0.010 g-P/day was only contained by the suspended biomass which produced 0.048 g-P/day in the permeate stream. Therefore, based on the phosphorus feeding rate, 27% and 72% were accumulated in the suspended sludge and sponge biomass in the BF-A/O-MBR system, respectively.

According to past research studies, extracellular polymeric substances (EPS) play a crucial role in the bio-absorption of pollutants [22,23]. Cloete and Oosthuizen [24] have reported that phosphorus removal in the activated sludge might not only be due to PAO activity but also can occur as a consequence of EPS acting as a phosphorus reservoir. The hypothesis model of Zhang et al. [25] suggests that under anaerobic conditions, long-chain polyphosphates are degraded to short-chain polyphosphates, pyrophosphates, and orthophosphates using the energy obtained from the glycogen degradation inside the PAO cells. The PAO cells then release most of the pyrophosphates and orthophosphates, while a part of the short-chain polyphosphates produced during this process were released into the EPS matrix. The polyphosphates and the pyrophosphates are mostly adsorbed by the EPS matrix, while the orthophosphates could easily pass through the EPS matrix. Based on the hypothesis model described above, some phosphate forms can be absorbed by EPS (e.g., polyphosphates and pyrophosphates) and the other forms are taken up by PAOs (e.g., orthophosphates). Therefore, phosphorus removal in the MBR can be achieved by retention of EPS and PAOs by membrane filtration.

Regarding EPS concentrations in this study (Table 1), statistically significant differences were observed between the BF-A/O-MBR and C-A/O-MBR systems, with average total EPS concentrations of 128.13 and 108.77 mg/gMLVSS, respectively. Moreover, the values of MLVSS concentrations were significantly higher in the BF-A/O-MBR system (10,910 mg/l) than in the C-A/O-MBR system (8,530 mg/l). Yigit et al. [26] reported that higher MLSS levels typically increase the concentration of EPS. Higher MLVSS concentrations caused higher levels of EPS to be secreted from the microorganism cells. In this regard, phosphorus accumulated in greater amounts inside the PAO cells and were absorbed in greater amounts by EPS, simultaneously. Consequently, through the contribution of the membrane separation process, all of the PAOs containing high amounts of phosphates were retained inside the reactor. In this way, a very high phosphorus removal rate was achieved in the BF-A/O-MBR system. Therefore, EPS might be one of the most important factors in the phosphate adsorption by way of suspended biomass and sponge biomass in the BF-A/O-MBR system. However, it remains unclear and information is still lacking that would explain what was truly happening in this phenomenon. The phenomenon would need to be further investigated in future work.

### Real-time quantitative PCR

**Fig 3:**
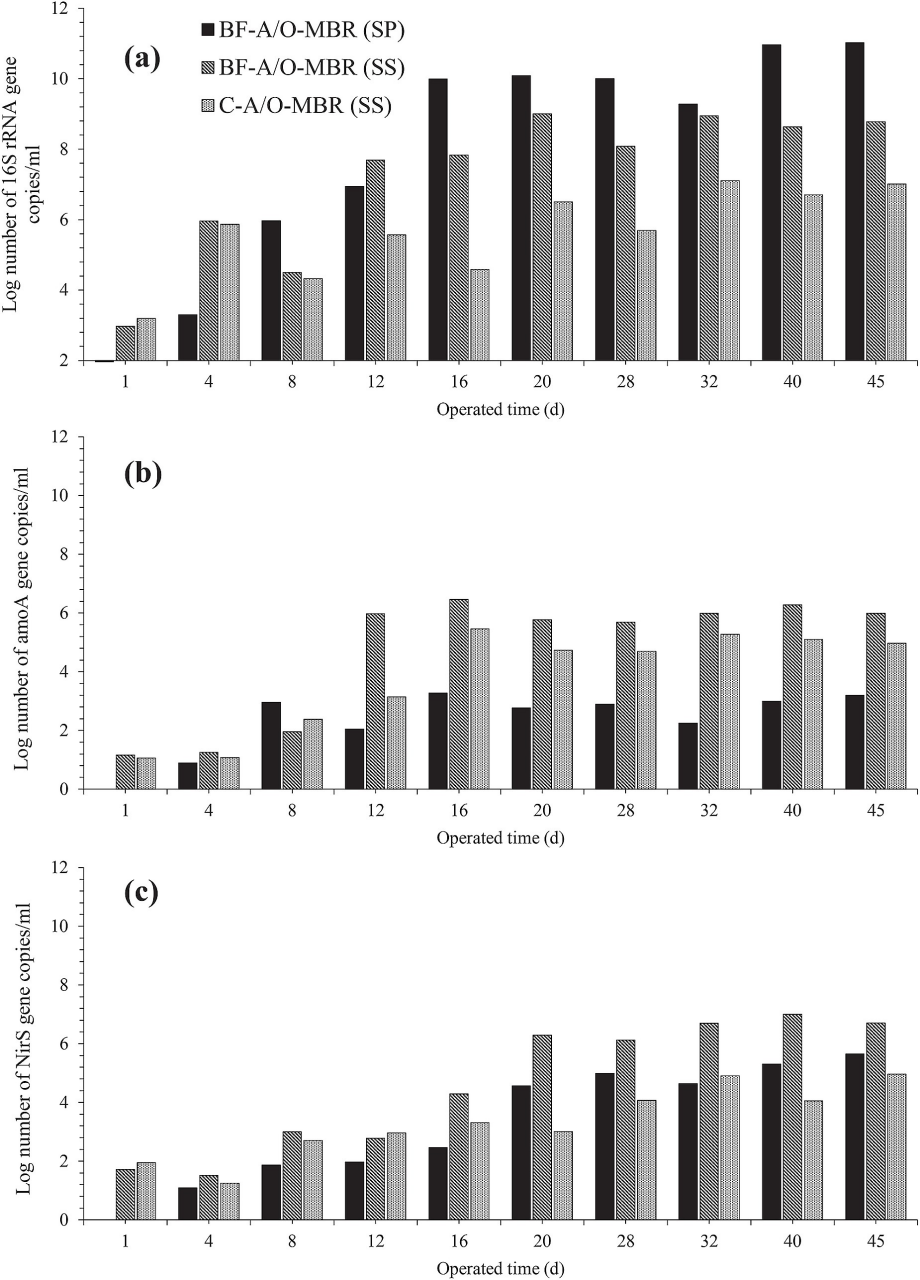
Bacterial 16S rRNA gene (a), AmoA gene (b) and NirS gene (c) copy number change with operating time of sponge biomass sample of BF-A/O-MBR system (BF-A/O-MBR (SP)) and suspended sludge samples of both BF-A/O-MBR and C-A/O-MBR system (BF-A/O-MBR (SS) and C-A/O-MBR (SS), respectively).

Quantitative real-time PCR of 16S rRNA genes, bacterial *amoA* genes, and *NirS* genes was applied to estimate the abundance of total bacteria, ammonium oxidizing bacteria (AOB) and denitrifying bacteria, respectively, in the sponge biomass samples (BF-A/O-MBR system) and the suspended sludge samples (BF-A/O-MBR and C-A/O-MBR system) (Fig 3 and Table 2). At the beginning of the experiment, the amounts of total bacteria, AOB and denitrifying bacteria of the suspended sludge samples in the BF-A/O-MBR system were started at approximately 9.3 x 10^2^, 1.4 x 10^1^ and 5.2 x 10^1^ copies/ml of sludge biomass, respectively. Meanwhile, the virgin sponge was implemented without any biomass accumulation. The amounts of total bacteria, AOB and denitrifying bacteria in both the sponge biomass and the suspended biomass samples gradually increased and fluctuated during the first 16 days of operation. A stabilization in abundance of total bacteria, AOB and denitrifying bacteria in both the sponge biomass and the suspended biomass samples were observed after day 20 of the operation. At the end of the experiment (day 45), total bacteria, AOB and denitrifying bacteria concentrations of the BF-A/O-MBR system were 1.1 x 10^11^, 1.6 x 10^3^ and 4.5 x 10^5^ copies/ml of the sponge biomass and 6.0 x 10^8^, 9.9 x 10^5^ and 5.1 x 10^6^ copies/ml of the sludge biomass, respectively. During the stabilization stage, the amounts of total bacteria in the sponge biomass were higher than that of the suspended sludge. However, for AOB and denitrifying bacteria, the concentrations were always lower in the sponge biomass when compared with the suspended sludge. The results clearly revealed that a compacted sponge at an adhesive stage could contain higher amounts of active biomass than at the suspended stage. Nevertheless, AOB and denitrifying bacteria likely preferred to grow under the suspended stage rather than under the adhesive stage. Due to the fact that the compacted sponge was added into the anoxic tank, the inner side of the compacted sponge may lack the oxygen and nitrate source that are used as an electron acceptor for AOB and denitrifying bacteria, respectively, resulting in lower amounts of these bacteria groups in the sponge biomass. Therefore, the majority of the nitrification and denitrification processes in this study tended to occur in the suspended sludge rather than in the sponge biomass.

**Table 2:** Quantitative real-time PCR results of 16S rRNA genes, bacterial amoA genes, NirS and the ratio relative to 16S rRNA gene copy number.

16S rRNA						
Day	BF-A/O-MBR (SP)		BF-A/O-MBR (SS)		C-A/O-MBR (SS)	
	copy/ml		copy/ml		copy/ml	
1	0		9.32 x 10^2^		1.55 x 10^3^	
4	2.01 x 10^3^		9.12 x 10^5^		7.32 x 10^5^	
8	9.32 x 10^5^		3.15 x 10^4^		2.11 x 10^4^	
12	8.83 x 10^6^		4.88 x 10^7^		3.69 x10^5^	
16	9.87 x 10^9^		6.78 x 10^7^		3.80 x 10^4^	
20	1.21 x 10^10^		9.99 x 10^8^		3.20 x 10^6^	
28	9.92 x 10^9^		1.19 x 10^8^		4.90 x 10^5^	
32	1.89 x 10^9^		8.90 x 10^8^		8.90 x 10^9^	
40	9.14 x 10^10^		4.31 x 10^8^		5.03 x 10^6^	
45	1.06 x 10^11^		6.04 x 10^8^		1.03 x 10^7^	
amoA						
Day	BF-A/O-MBR (SP)		BF-A/O-MBR (SS)		C-A/O-MBR (SS)	
	copy/ml	amoA/16S ratio (%)	copy/ml	amoA/16S ratio (%)	copy/ml	amoA/16S ratio (%)
1	0	0.00	1.45 x 10^1^	1.56	1.15 x 10^1^	0.74
4	0.78 x 10^1^	0.39	1.79 x 10^1^	0.00	1.19 x 10^1^	0.00
8	9.10 x 10^2^	0.10	9.00 x 10^1^	0.29	2.40 x 10^2^	1.14
12	1.10 x 10^2^	< 0.1	9.40 x 10^5^	1.93	1.40 x 10^3^	0.38
16	1.88 x 10^3^	< 0.1	2.88 x 10^6^	4.25	1.88 x 10^3^	7.59
20	5.84 x 10^2^	< 0.1	5.84 x 10^5^	0.06	5.38 x 10^4^	1.68
28	7.82 x 10^2^	< 0.1	4.84 x 10^5^	0.41	4.88 x 10^4^	9.96
32	1.79 x 10^2^	< 0.1	9.88 x 10^5^	0.11	1.88 x 10^5^	1.48
40	9.92 x 10^2^	< 0.1	1.88 x 10^6^	0.44	1.24 x 10^5^	2.47
45	1.59 x 10^3^	< 0.1	9.88 x 10^5^	0.16	9.24 x 10^4^	0.90
NirS						
Day	BF-A/O-MBR (SP)		BF-A/O-MBR (SS)		C-A/O-MBR (SS)	
	copy/ml	NirS/16S ratio (%)	copy/ml	NirS/16S ratio (%)	copy/ml	NirS/16S ratio (%)
1	0	0.00	5.22 x 10^1^	5.60	8.76 x 10^1^	5.65
4	1.22 x 10^1^	0.61	3.22 x 10^1^	< 0.1	1.76 x 10^1^	< 0.1
8	7.22 x 10^1^	0.01	9.92 x 10^2^	3.15	5.08 x 10^2^	2.41
12	9.22 x 10^1^	< 0.1	5.92 x 10^2^	< 0.1	9.08 x 10^2^	0.25
16	2.92 x 10^2^	< 0.1	1.93 x 10^4^	0.03	2.04 x 10^3^	5.36
20	3.63 x 10^4^	< 0.1	1.94 x 10^6^	0.19	1.02 x 10^3^	0.03
28	9.67 x 10^4^	< 0.1	1.34 x 10^6^	1.12	1.17 x 10^4^	2.40
32	4.36 x 10^4^	< 0.1	4.99 x 10^6^	0.56	8.11 x 10^4^	0.64
40	2.00 x 10^5^	< 0.1	9.94 x 10^6^	2.31	1.11 x 10^4^	0.22
45	4.51 x 10^5^	< 0.1	5.09 x 10^6^	0.84	9.18 x 10^4^	0.89

When comparing the abundance of the bacteria groups of the two systems (BF-A/O-MBR and C-A/O-MBR systems), it was found that after inoculation, the averages of total bacteria, AOB and denitrifying bacteria of the suspended sludge samples were approximately 1.7 x 10^3^ ± 114, 1.3 x 10^1^ ± 42 and 7.0 x 10^1^ ± 12 copies/ml of the sludge biomass, respectively, in the two reactors. There was no significant difference between the two reactors over the first 8 days of operation (p = 0.05). After day 16, the amounts of total bacteria, AOB and denitrifying bacteria were all higher in the BF-A/O-MBR system. The amounts of total bacteria, AOB and denitrifying bacteria distinctly increased during day 20 in both systems and seemed to be constant after that time. The results suggested that the specific amounts of microorganisms that were chosen for their capability to degrade the chemical components of synthetic wastewater would significantly achieve the stability and performance of both systems after 20 days. At day 45, the concentrations of total bacteria, AOB and denitrifying bacteria were 6.4 x 10^8^, 1.8 x 10^6^ and 5.1 x 10^6^ copies/ml of sludge biomass for the BF-A/O-MBR system and 1.2 x 10^7^, 1.2 x 10^5^ and 9.1 x 10^4^ copies/ml of sludge biomass for the C-A/O-MBR system, respectively. Obviously, the sponge biomass played an essential role in the growth of bacteria in the suspended sludge. Therefore, it could be concluded that the significant improvement of TN removal in the BF-A/O-MBR system, when compared with the C-A/O-MBR system, was achieved mainly by adding a sponge to the anoxic tank.

### Richness and diversity of bacteria community

Diversity indices serve as a valuable tool to quantify diversity in a population and describe its numerical structure, by combining richness and evenness components. Two diversity indices, the Shannon index (H’) and the reciprocal of Simpson’s index (D), were used to assess the bacterial community diversity in this study. The diversity index results of the sponge biomass sample in the BF-A/O-MBR system and suspended sludge samples in the BF-A/O-MBR and C-A/O-MBR systems are shown in Table 3. Both the Shannon and 1/Simpson indexes of 4.78 and 37.72 in the sponge biomass sample showed higher diversity values than those of the suspended sludge sample of the BF-A/O-MBR system at 4.36 and 36.98, respectively. Moreover, the Shannon and 1/Simpson indexes of both the sponge biomass and suspended sludge samples in the BF-A/O-MBR system were significantly higher than the C-A/O-MBR system (3.95 and 24.93, respectively). The results showed that the sponge biomass as an active attached biomass was more diverse than the suspended sludge. This finding also revealed that the existence of the compacted sponge in the anoxic tank had an effect on the diversity of the microbial community in the suspended sludge. The reason for this is that when the compacted sponge was constructed in the anoxic tank, the biofilm which developed on the sponge surface became thick and eventually sloughed off to the suspended sludge. Therefore, some of the microorganisms that attached and grew on the sponge surface were exposed to the suspended sludge, which led to a high level of diversity in the suspended sludge.

**Table 3:** Richness and diversity index of bacteria community in the sponge biomass sample of BF-A/O-MBR system (BF-A/O-MBR (SP)) and suspended sludge samples of both BF-A/O-MBR and C-A/O-MBR system (BF-A/O-MBR (SS) and C-A/O-MBR (SS), respectively).

Sample ID	BF-A/O-MBR (SP)	BF-A/O-MBR (SS)	C-A/O-MBR (SS)
Observed OTU	671	623	579
Shannon index (H')	4.78	4.36	3.95
Species richness (Hmax)	6.50	6.43	6.36
Species evenness (E)	0.69	0.68	0.62
1/Simpson index	37.72	36.98	24.93

**Fig 4:**
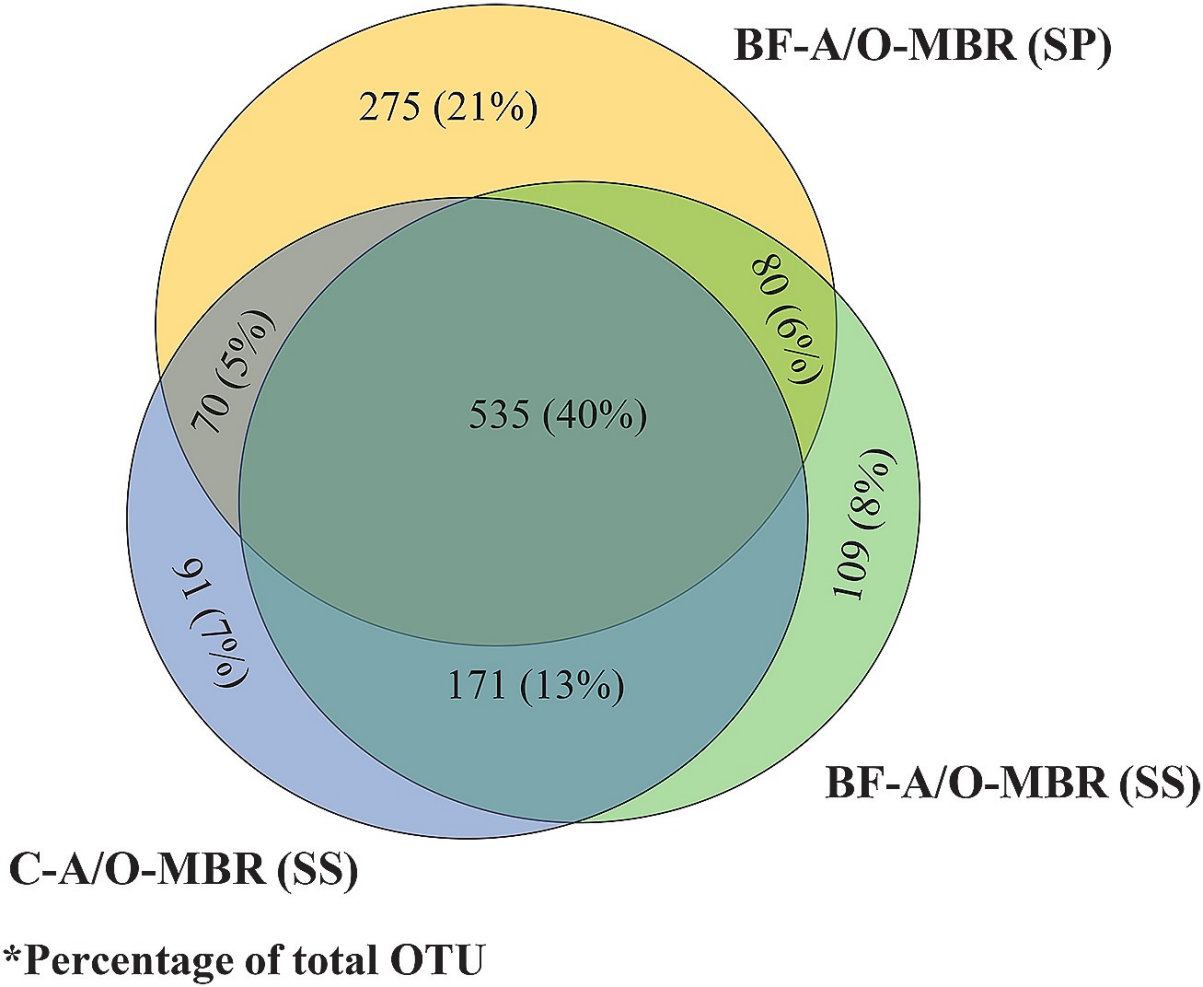
Venn’s diagram of exclusive and shared OTU between sponge biomass sample of BF-A/O-MBR system (BF-A/O-MBR (SP)) and suspended sludge samples of both BF-A/O-MBR and C-A/O-MBR system (BF-A/O-MBR (SS) and C-A/O-MBR (SS), respectively).

A Venn diagram of the exclusive and shared OTU (genus level) values of the sponge biomass of the BF-A/O-MBR system and suspended sludge samples of both the BF-A/O-MBR and C-A/O-MBR systems is presented in Fig 4. There were 535 OTUs seen in three samples, while 275, 109 and 91 OTUs were unique for the sponge biomass and suspended sludge samples of the BF-A/O-MBR system and the suspended sludge sample of the C-A/O-MBR system, respectively. The differences of unique OTUs in the three samples indicated that the communities in the sponge biomass and the suspended sludge of the system with compacted sponge samples (BF-A/O-MBR system) were more diverse than those in the suspended sludge of the system without a compacted sponge (C-A/O-MBR system).

Therefore, it could be concluded that the presence of a compacted sponge in the anoxic tank of the BF-A/O-MBR system may contribute to high levels of nitrogen and phosphorus removal performance (87.79% and 95.3%, respectively), which is reflected in the higher presence of microbial diversity in the system. Some of the species (such as nitrifiers, denitrifiers and PAOs) that appeared in the BF-A/O-MBR system and disappeared in the C-A/O-MBR system might be the main microorganisms that are involved in the nutrient removal mechanisms in this study.

### Comparison of bacterial communities

**Fig 5:**
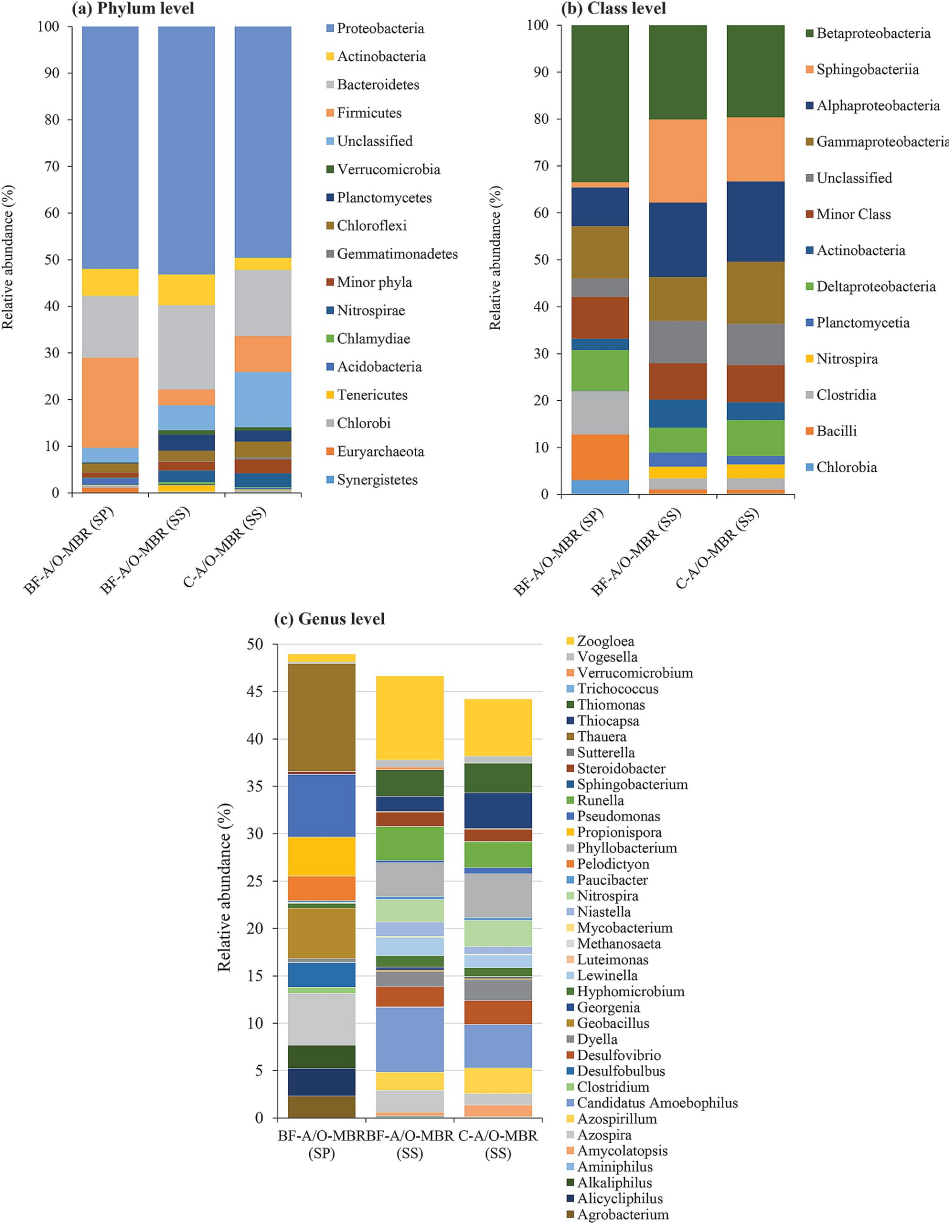
Relative abundance of bacterial phyla level (a), class level (b) and genus level (c) in the sponge biomass sample of BF-A/O-MBR system (BF-A/O-MBR (SP)) and suspended sludge samples of both BF-A/O-MBR and C-A/O-MBR system (BF-A/O-MBR (SS) and C-A/O-MBR (SS), respectively).

Phyla distributions describing microbial diversity in the BF-A/O-MBR system (sponge biomass and suspended sludge samples) and the C-A/O-MBR system (suspended sludge sample) are summarized in Fig 5(A) and S5 Table. A total of 30 phyla were observed in the DNA of the sponge biomass sample of the BF-A/O-MBR system, and 29 phyla were observed in the DNA of the suspended sludge samples of both the BF-A/O-MBR and C-A/O-MBR systems. The term ‘unclassified’ is used to refer to the sequences that could not be classified up to the phylum level. Phyla that were observed at less than 2% average abundance were grouped in ‘Minor phyla’. High proportions of unclassified sequences in a study applying illumina sequencing have been previously reported [27,28]. In this study, depending on the samples, the proportions of the unclassified phyla sequences were 3.06% in the sponge biomass of the BF-A/O-MBR system and, 5.28 and 11.78% in the suspended sludge of the BF-A/O-MBR and C-A/O-MBR systems, respectively.

According to the sequencing data, about half of the total bacteria in the sponge biomass sample of the BF-A/O-MBR system and the suspended sludge samples of the BF-A/O-MBR and C-A/O-MBR systems belonged to Proteobacteria (51.94, 53.16 and 49.58%, respectively). The results revealed a similar predominant population at the phylum level with other research studies [29,30]. Proteobacteria, a group of Gram-negative bacteria, were found to easily attach themselves to the surface of the media due to the fact that the major components of their outer surface include lipopolysaccharides [31]. Tang et al. [32] mentioned that an adhesive phase was a more suitable environment for the growth of Proteobacteria than that of a suspended phase. Therefore, the sponge could promote the growth of these phylum bacteria in the reactor, which was the main reason why Proteobacteria overwhelmed a larger proportion in both the sponge biomass and the suspended sludge of the BF-A/O-MBR system. The levels of abundance of Bacteroidetes found in the sponge biomass and suspended sludge samples of the BF-A/O-MBR system were 13.22 and 18.06%, respectively, while 14.16% was found in the suspended sludge sample of the C-A/O-MBR system. Bacteroidetes play crucial roles in degrading organic matter and can potentially release proteinaceous EPS [33]. Thus, a higher abundance of Bacteroidetes in the BF-A/O-MBR system is expected to cause the accumulation of biopolymers in the mixed liquor. This finding is consistent with the higher levels of total EPS concentrations found in the BF-A/O-MBR system (Table 1).

A very high abundance of Firmicutes was found in the sponge biomass sample (19.35%), while only 3.43% was observed in the suspended sludge sample of the BF-A/O-MBR system. In comparison with the C-A/O-MBR system, the sponge biomass appeared to trigger a decrease in the relative proportion of Firmicutes in the suspended sludge of the BF-A/O-MBR system, since the higher abundance of this phylum was observed in the suspended sludge sample of the C-A/O-MBR system (7.72%). It was found that Firmicutes were the main factors that influenced membrane fouling in the MBR system [34]. Moreover, the enrichment of filamentous Chloroflexi in the sponge biomass and suspended sludge samples of the BF-A/O-MBR system (1.87 and 2.29%, respectively) were less than in the suspended sludge sample of the C-A/O-MBR system (3.48%). A dominance of filamentous bacteria can promote filamentous bulking in the suspended sludge. Filamentous bulking can also significantly increase the production of SMPs, which in turn substantially increase the fouling of membranes [35]. When considered with the TMP profiles (S1 Fig), it was found that the fouling rate in the BF-A/O-MBR system (2.60 kPa/day) was almost two times lower than that of the C-A/O-MBR system (4.24 kPa/day). The findings clearly indicate that the sponge addition in the anoxic tank seems to prolong membrane cycle by reducing Firmicutes and filamentous Chloroflexi bacteria in the suspended sludge. In this regard, Firmicutes and filamentous Chloroflexi were more attracted to the sponge surface than the membrane surface.

It was found that the sponge biomass and suspended sludge samples of the BF-A/O-MBR system contained a population of Actinobacteria which accounted for 5.89% and 6.60%, respectively, while in the suspended sludge sample of the C-A/O-MBR system, the figure was 2.62%. Beer et al. [36] demonstrated that Actinobacteria are important in the biological phosphorus removal processes, due to the ability of anaerobic substrate assimilation and the subsequent aerobic phosphate assimilation and capability of poly-P storage. These results suggest that the BF-A/O-MBR system could achieve higher TP removal efficiency levels when compared with the C-A/O-MBR system by promoting the growth of Actinobacteria in the system. The anaerobic area was created inside the sponge due to the decreasing trend of DO concentration from the surface to the inside of the sponge. Therefore, Actinobacteria may grow in the deep zone of the sponge biomass. When the sponge biomass became thick and was then sloughed off, it could also lead to an increased population of Actinobacteria in the suspended sludge. Nitrospirae was also detected in the suspended sludge samples at a proportion of 2.50 and 2.97% in the BF-A/O-MBR and C-A/O-MBR systems, respectively. However, only 0.06% was observed in the sponge biomass sample. The Nitrospirae bacterium, rather than Nitrobacter, was able to oxidize NO_2_-N into NO_3_-N as has been described in a recent research study [37]. The stable proportion of this phyla might explain why higher and more stable nitrification performance occurred in both systems. A higher abundance of phylum Nitrospirae found in the suspended sludge rather than in the sponge biomass and the qPCR results indicated that nitrification tended to happen at the outer side rather than on the inner side of the sponge biomass.

At the class level, Betaproteobacteria (33.45, 20.08 and 19.58%), Alphaproteobacteria (8.31, 15.86 and 17.10%), Gammaproteobacteria (11.11, 9.36 and 13.28%) and Deltaproteobacteria (8.65, 5.28 and 7.64%) were the most abundance classes in all samples (sponge biomass sample of the BF-A/O-MBR system and suspended sludge samples of the BF-A/O-MBR and C-A/O-MBR systems, respectively) (Fig 5(B) and S6 Table). Betaproteobacteria, the most abundant class, is largely responsible for organic and nutrient removal [38]. Betaproteobacteria, Alphaproteobacteria, Gammaproteobacteria, and Deltaproteobacteria are also well-known for being present in butyrate, glucose, propionate, and acetate-utilizing microbial populations [39]. Alphaproteobacteria, which are a dominant class in some samples of biofilm, are responsible for the biodegradation of some micro-pollutants, i.e., nitrogen and COD removal [40]. The high nitrogen removal in the BF-A/O-MBR and C-A/O-MBR systems (>80%) might be a response of this bacteria group becoming enriched in both systems. Gammaproteobacteria have been shown to favorably adhere to membrane surfaces when compared to those of other microorganisms [41]. A higher relative abundance of Gammaproteobacteria in the suspended sludge sample in the C-A/O-MBR system might be partly responsible for severe membrane bio fouling in the system when compared to the BF-A/O-MBR system.

The relative abundance of Nitrospira and Clostridia was similar in the suspended sludge samples of the BF-A/O-MBR system (2.50 and 2.37%) and C-A/O-MBR system (2.97 and 2.43%), respectively. However, while Nitrospira was very low (0.05%), Clostridia revealed higher abundance (9.13%) in the sponge biomass sample of the BF-A/O-MBR system. Nitrospira plays a pivotal role in nitrification as an aerobic chemolithoautotrophic nitrite-oxidizing bacterium. Nitrospira belongs to the nitrite oxidizing bacteria category, and Nitrospira-like bacteria have been reported as dominant nitrite oxidizers in biofilms that were collected from wastewater treatment plants [42]. Nitrospira and similar bacteria are slow-growing organisms which may be favored for long SRT in this study. Therefore, the suspended sludge might contribute to establishing favorable growth conditions for Nitrospira-like bacteria rather that the sponge biomass, thereby partly contributing to ammonia oxidation in the MBR. As for Clostridia, they are well known to be electrochemically active and capable of fixing N_2_ [43].

**Fig 6:**
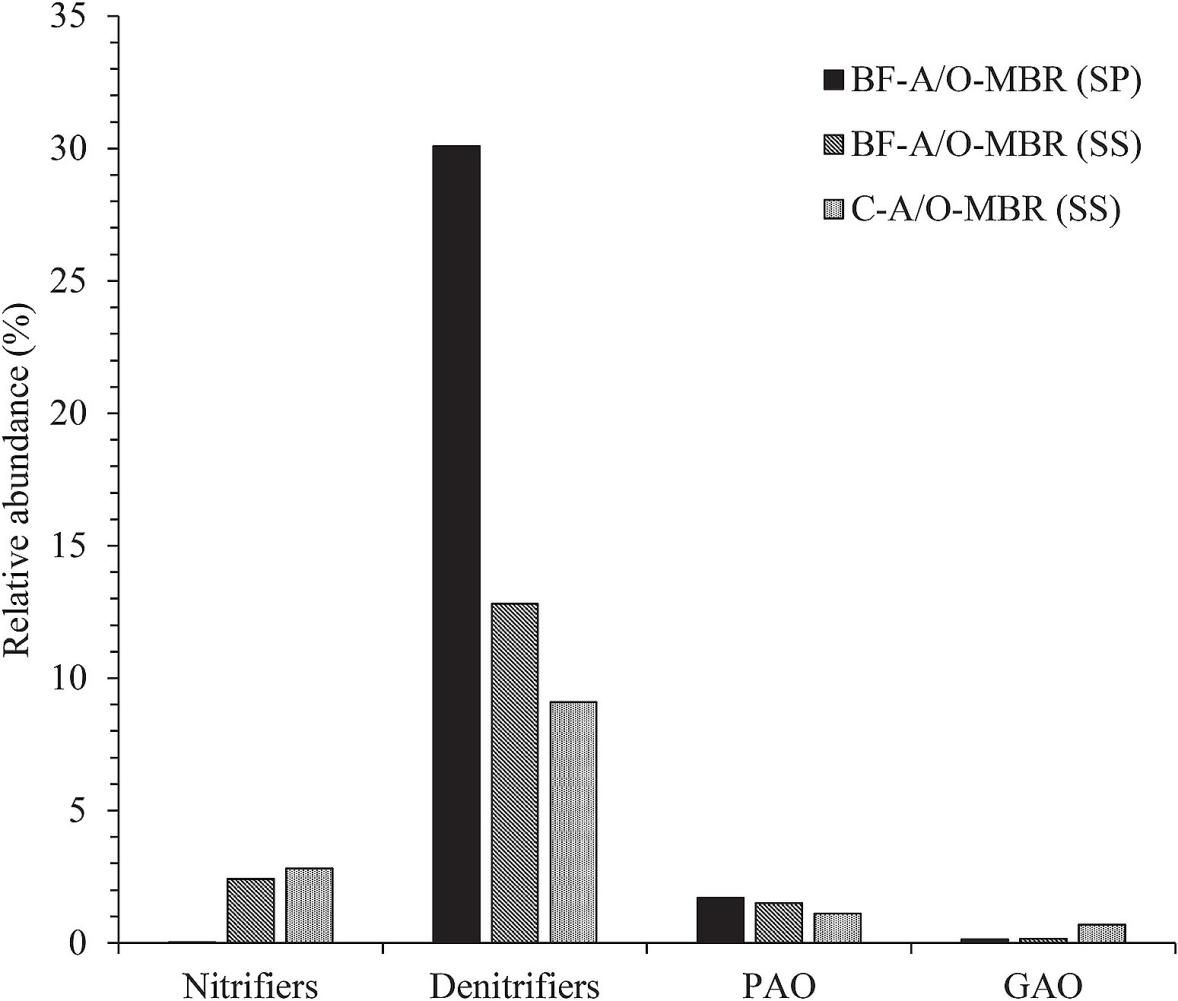
Relative abundance of potential functional bacterial groups including: Nitrifiers (*Nitrospira*), denitrifiers (*Azospira*, *Geobacillus*, *Hyphomicrobium*, *Pseudomonas*, *Thauera* and *Zoogloea*), polyphosphate accumulating organisms (PAO: *Dechloromonas*) and glycogen accumulating organisms (GAO: *Sphingomonas* and *Amaricoccus*).

A close investigation at the genus level (Fig 5(B) and S7 Table) showed that the genus *Nitrospira*, which is defined as nitrite oxidizing bacteria (NOB) [37], was detected in the suspended sludge samples at proportions of 2.40 and 2.80% in the BF-A/O-MBR and C-A/O-MBR systems, respectively. However, only 0.02% was found in the sponge biomass sample of the BF-A/O-MBR system. Obviously, nitrifiers are likely to prefer a suspended stage rather than an adhesive stage. However, in both systems, the nitrification process was nearly complete and most of the influent NH_4_^+^-N entering the aerobic MBR tank were oxidized entirely into NO_3_^−^N regardless of whether or not a sponge was added. Moreover, the extremely long SRT applied in this study was advantageous for slow-growing bacteria such as nitrifying bacteria [44]. This finding revealed that most of the nitrification process tended to occur outside the sponge biomass and that the existence of a compacted sponge was not involved in the ammonia removal process that occurred in this study. Additionally, sequencing analysis showed that only a small amount of nitrifiers (Fig 6) were detected in the reactor when compared with the qPCR results on the same day, as has been described above (Fig 3). The results suggested that real-time PCR using a functional gene is an effective tool for quantitative targeting of the bacteria group rather than illumina sequencing.

As for denitrifying the bacteria population, six genera were detected in all samples including *Azospira* [45], *Geobacillus* [46], *Hyphomicrobium* [47], *Pseudomonas* [48], *Thauera* [49] and *Zoogloea* [44]. As is shown in Fig 6, the highest level of abundance of denitrifying bacteria was found in the sponge biomass sample of the BF-A/O-MBR system, accounting for 30.10%. However, they accounted for a lower proportion in the suspended sludge sample of the BF-A/O-MBR and C-A/O-MBR systems (12.08 and 9.09%, respectively). The highest genus in the sponge biomass sample was *Thauera* (11.40%) followed by *Pseudomonas* (6.59%), *Azospira* (5.43%) and *Geobacillus* (5.33%), while the highest genus in the suspended sludge samples of the BF-A/O-MBR and C-A/O-MBR systems was *Zoogloea* (8.86 and 6.02%, respectively) followed by *Azospira* (2.32 and 1.20%, respectively) and *Hyphomicrobium* (1.18 and 0.94%, respectively). The deficiency of denitrifying bacteria in the C-A/O-MBR system can be a reflex of the significantly lower value of TN removal efficiency (p = 0.05). A significantly higher abundance of denitrifying bacteria in the sponge biomass sample revealed that the presence of a compacted sponge in the BF-A/O-MBR system could improve TN removal by promoting the growth of some of denitrifying bacteria groups in the reactor.

*Dechloromonas*, which have the ability to store polyphosphates (PAO) in full-scale wastewater treatment plants [50], were found to be predominant in both the sponge biomass and suspended sludge samples of the BF-A/O-MBR system (1.71 and 1.51%, respectively) rather than the suspended sludge samples of the C-A/O-MBR system (1.10%). *Sphingomonas* and *Amaricoccus* related glycogen accumulating organisms (GAOs) [51,52] were also detected in all samples. The abundance of these two genera was found in the suspended sludge sample of the C-A/O-MBR system (0.44 and 0.25%, respectively). However, amounts lower than 0.1% were observed in both the sponge biomass and the suspended sludge sample of the BF-A/O-MBR system. As has been illustrated in Fig 6, PAOs and GAOs seemed to compete with each other. Numerous studies have shown that GAOs and PAOs compete for the same substrate under anaerobic conditions, but no accumulation of polyphosphates occurred under aerobic conditions [53]. These results indicate that the absence of a sponge biomass inhibited the growth of PAOs and increased the abundance of GAOs resulting in the deterioration of the phosphorus removal performance. Moreover, this finding also confirmed the hypothesis that the sponge media could provide an anaerobic environment under anoxic conditions for PAOs to consume a carbon source and release phosphates at the same time, resulting in a high degree of phosphorus removal that was achieved even without the anaerobic compartment.

**Fig 7:**
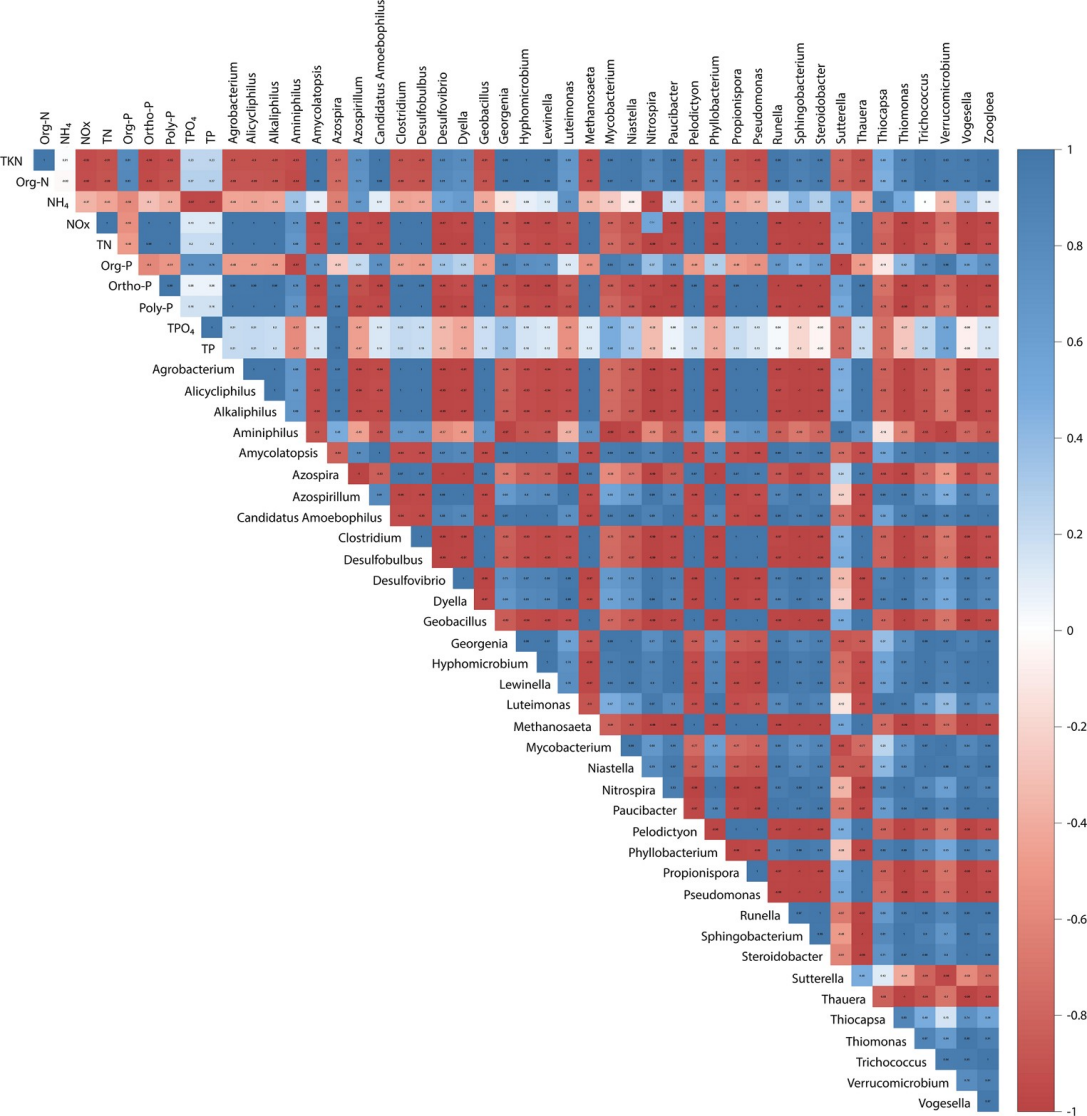
Heatmap of cluster analysis of the most abundant genera (>2% of the total OTU reads in each group) for sponge biomass sample of BF-A/O-MBR system (BF-A/O-MBR (SP)) and suspended sludge samples of both BF-A/O-MBR and C-A/O-MBR system (BF-A/O-MBR (SS) and C-A/O-MBR (SS), respectively) (b).

To obtain a higher resolution of community composition, a heatmap was utilized to illustrate the relative abundance of the 37 OTUs found in the three samples presented in Fig 7. Three major groups were identified and were separated by only six units in the bacterial community pattern cluster analysis, indicating clear distinctions of microbial community structure due to the different stages that exist (adhesive and suspended stage) and with and without the addition of a compacted sponge. As is shown in Fig 7, clusters 1 and 2, which were comprised of *Azospirillum*, *Desulfovibrio*, *Nitrospira*, *Phyllobacterium*, *Runella*, *Thiocapsa*, *Thiomonas*, *Dyella*, *Hyphomicrobium*, *Lewinella*, *Niastella*, *Steroidobacter* and *Vogesella*, were dominant in the suspended sludge sample (BF-A/O-MBR (SS) and C-A/O-MBR (SS)) of both systems. Most of them belong to phyla Proteobacteria and Bacteroidetes which are commonly found in activated sludge and biofilm. The heatmap clearly shows that the existence of the compacted sponge in the BF-A/O-MBR system could promote the growth of *Zoogloea*, *Candidatus Amoebophilus* and *Azospira* (cluster 5) in the suspended sludge. A significant difference in the microbial community was observed in the sponge biomass sample of the BF-A/O-MBR system (BF-A/O-MBR (SP)). The levels of abundance of clusters 1 and 2 decreased in the suspended sludge samples, while levels in clusters 4 and 6 were more dominant in the sponge biomass samples of the BF-A/O-MBR system. *Thauera*, *Propionispora*, *Geobacillus*, *Pseudomonas*, and *Azospira* were found to be distinctly present in the sponge biomass sample. Due to the denitrification capacities of *Zoogloea*, *Azospira*, *Thauera*, *Geobacillus* and *Pseudomonas*, which were dominant in the BF-A/O-MBR system. This would indicate that the significantly higher level of TN removal that was found in this system was encouraged by the presence of the compacted sponge inside the anoxic tank.

### Correlation between nutrient removal and bacterial community

**Fig 8:**
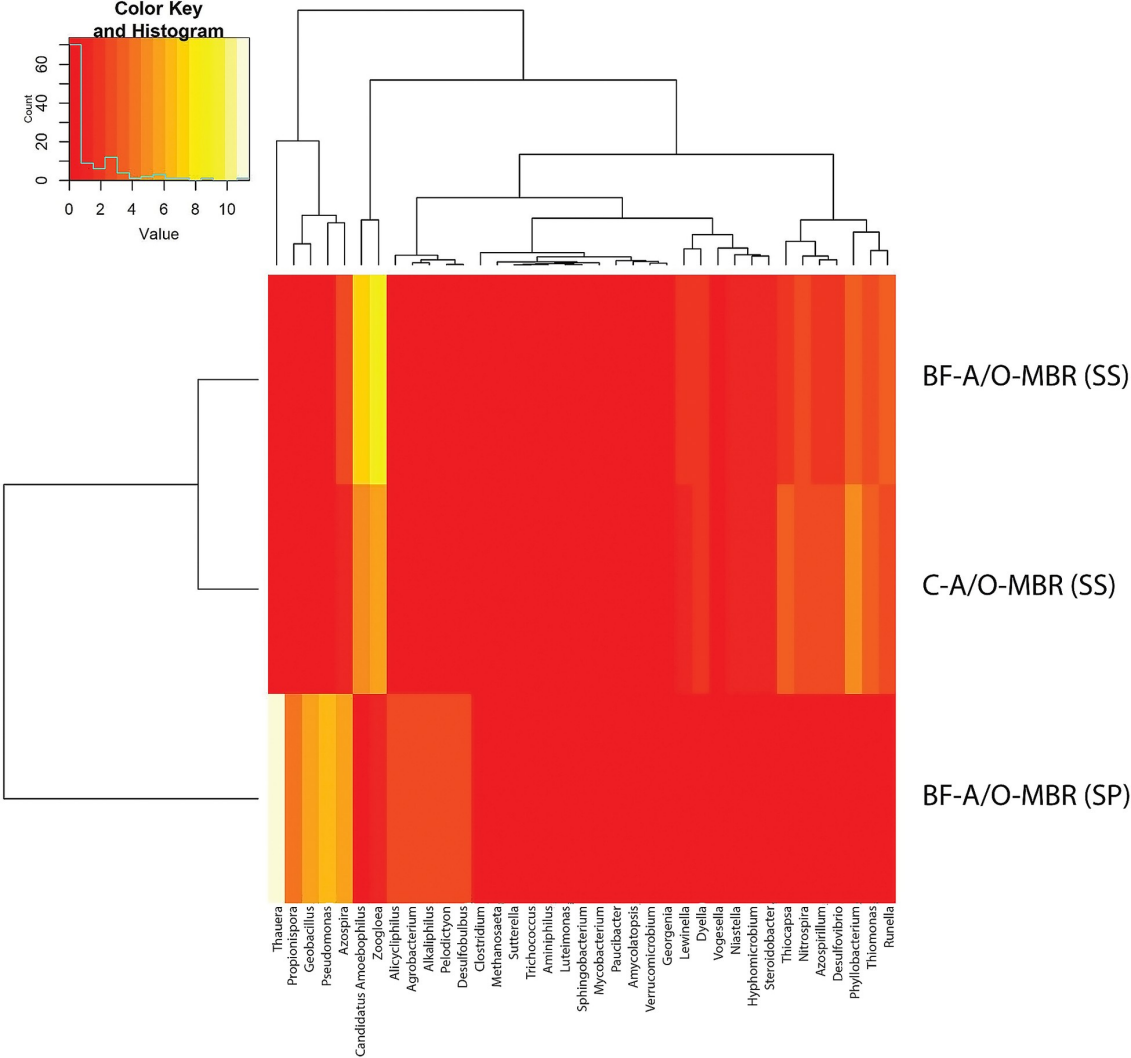
Heatmap of Pearson’s correlation between the most abundant bacteria (>2% of the total OTU reads in each group) and nutrient concentrations in the system.

To investigate the ecological correlation between the bacterial community composition (at the genus level), and nitrogen removal, Pearson’s correlation was conducted using TKN, organic-nitrogen (Org-N), ammonia-nitrogen (NH_4_), nitrite-nitrogen + nitrate-nitrogen (NOx) and total nitrogen (TN) concentrations in different zones (anoxic tank and MBR tank) as key variables (Fig 8). A heatmap of Pearson’s correlation showed that the genus *Amycolatopsis* (r_p_ = -0.94), *Azospirillum* (r_p_ = -0.96), *Candidatus Amoebophilus* (r_p_ = -0.94), *Desulfovibrio* (r_p_ = -0.99), *Dyella* (r_p_ = -0.97), *Georgenia* (r_p_ = -0.84), *Hyphomicrobium* (r_p_ = -0.94), *Lewinella* (r_p_ = -0.95), *Luteimonas* (r_p_ = -0.93), *Nitrospira* (r_p_ = -0.99), *Paucibacter* (r_p_ = -0.97), *Phyllobacterium* (r_p_ = -0.98), *Runella* (r_p_ = -0.97), *Sphingobacterium* (r_p_ = -1.00), *Steroidobacter* (r_p_ = -0.99), *Thiocapsa* (r_p_ = -0.81), *Thiomonas* (r_p_ = -1.00), *Trichococcus* (r_p_ = -0.90), *Vogesella* (r_p_ = -0.99) and *Zoogloea* (r_p_ = -0.94) contributed to the reduction of total nitrogen in the system due to a strong negative correlation with total nitrogen concentration. The genus *Nitrospira* was closely related to total nitrogen removal in the system owing to nitrification, while *Hyphomicrobium* and *Zoogloea* were assigned to total nitrogen removal in the system owing to denitrification. Therefore, it could be concluded that among the six denitrifying bacteria (i.e., *Azospira*, *Geobacillus*, *Hyphomicrobium*, *Pseudomonas*, *Thauera* and *Zoogloea*) detected in this study, *Hyphomicrobium* and *Zoogloea* were the most prevalent genera and that this outcome was related to the denitrification process. *Hyphomicrobium* and *Zoogloea* were most abundant in the suspended sludge samples of the BF-A/O-MBR system, followed by the suspended sludge sample of the C-A/O-MBR system. However, they were found to be lacking in the sponge biomass sample of the BF-A/O-MBR system. This finding clearly revealed that denitrifying bacteria, which display an important ability in the denitrifying process, preferred a suspended stage rather than an adhesive stage for growth. However, the presence of a compacted sponge in the BF-A/O-MBR system seemed to promote the growth of denitrifying bacteria when compared with the conventional C-A/O-MBR system. As for other genera, the findings are still unclear due to a lack of information regarding these genera and their relationship to nitrogen removal. The process of nitrogen removal could occur by simultaneous biomass assimilation and dissimilation (nitrification-denitrification process), and a possible reason for this is that these bacteria genera may contribute to total nitrogen removal by cell assimilation.

A heatmap of Pearson’s correlations between bacterial community composition (at the genus level) and phosphate forms is also presented in Fig 8. The results show that the correlation coefficient values (r_p_) between *Azospira* and polyphosphates, and total phosphates were found to be 0.96 and 0.98, respectively, which indicated that the impact of the genus *Azospira* had a strong positive correlation with poly-phosphate and total phosphate levels. Although several reports have identified the genus *Azospira* as a denitrifying bacterium in wastewater treatment plants [45, 54], some species belonging to the genus *Azospira* have been recently identified and are also considered to be potential PAOs [55]. The abundance of this genus coupled with an increase in phosphate concentrations in the reactor (positive correlation) indicated that *Azospira* might be associated with phosphate release in the sponge. However, phosphate uptake might be achieved by the presence of a number of other microorganisms that could not be sequenced by illumina sequencing in this study. Therefore, *Azospira*, which revealed higher levels of abundance in the sponge biomass (5.43%) and suspended sludge (2.32%) of the BF-A/O-MBR system when compared with the C-A/O-MBR system (1.20%), seemed to play a role in phosphorus removal in this study. On the contrary, *Thiocapsa* was found to have a significantly negative correlation with orthophosphate (r_p_ = -0.73), poly-phosphate (r_p_ = -0.79) and total phosphate (r_p_ = -0.73) concentrations. This indicated that as the abundance of the genus *Thiocapsa* increased, lower levels of orthophosphate, poly-phosphate and total phosphate concentrations accumulated in the reactor. However, the investigation is not fully conclusive and there is a lack of information that can indicate what the main mechanism of phosphorus is in the BF-A/O-MBR system when operated with complete sludge retention. Consequently, further studies need to be performed to clearly understand the mechanisms of this phenomenon.

## Conclusions

Two A/O-MBR systems (BF-A/O-MBR and C-A/O-MBR) were operated in parallel to compare system performance and microbial community composition. High average removal of COD, NH_4_^+^-N and TN was achieved in both systems. However, TP removal efficiency was remarkably higher in BF-A/O-MBR when compared with the C-A/O-MBR. TP mass balance suggested that under complete sludge retention, sponges play key roles in phosphorus release and accumulation. Results of sequencing clearly reveal that a compacted sponge in BF-A/O-MBR could promote the growth of bacteria involved in nutrient removal and reduce the growth of filamentous and bacteria related to membrane fouling in the suspended sludge.

## Supporting information

S1 Table Average DNA concentration extracted from sponge biomass and suspended sludge of BF-A/O-MBR and suspended sludge in C-A/O-MBR system. (DOCX)

S2 Table Oligonucleotide primers used for amplification. (DOCX)

S3 Table Reagents mixture composition for qPCR method: SYBR green 1. (DOCX)

S4 Table Phosphorus mass balance analysis in BF-A/O-MBR and C-A/O-MBR system. (DOCX)

S5 Table Relative abundance of bacterial at phyla level. (DOCX)

S6 Table Relative abundance of bacterial at class level. (DOCX)

S7 Table Relative abundance of bacterial at genus level. (DOCX)

S1 Fig Trans-membrane pressure (TMP) profiles of the A/O MBR systems. (TIF)
