# Supplementary material for: A simple way to improve a conventional A/O-MBR for high simultaneous carbon and nutrient removal from synthetic municipal wastewater
Source: PLoS One. 2019 Nov 22;14(11):e0214976. doi: 10.1371/journal.pone.0214976 (PMC6913871; doi:10.1371/journal.pone.0214976)
Supplement: S1 Table — (DOCX) [file pone.0214976.s001.docx]

**1S Table.** Average DNA concentration extracted from sponge biomass and suspended sludge of BF-A/O-MBR and suspended sludge in C-A/O-MBR system.

| **Day** | **DNA concentration (ng/µL)** | | |
| --- | --- | --- | --- |
|  | **BF-A/O-MBR(SP)** | **BF-A/O-MBR(SS)** | **C-A/O-MBR(SS)** |
| 1 | - | 17.7 ± 1.4 | 29.5 ± 2.1 |
| 4 | 11.6 ± 1.5 | 28.4 ± 4.2 | 21.3 ± 10.9 |
| 8 | 27.0 ± 7.4 | 67.1 ± 11.7 | 32.7 ± 6.9 |
| 12 | 42.3 ± 5.9 | 32.9 ± 20.9 | 60.4 ± 27.8 |
| 16 | 77.1 ± 19.4 | 65.4 ± 28.1 | 41.7 ± 16.4 |
| 20 | 129.7 ± 7.5 | 87.5 ± 14.2 | 61,6 ± 11.1 |
| 28 | 90.6 ± 11.5 | 78.4 ± 28.0 | 87.2 ± 17.3 |
| 32 | 149.1 ± 21.7 | 105.7 ± 32.8 | 83.6 ± 32.1 |
| 40 | 107.2 ± 7.8 | 90.4 ± 5.6 | 64.9 ± 21.9 |
| 45 | 144.7 ± 28.5 | 98.1 ± 10.9 | 105.9 ± 45.3 |
