# Supplementary material for: A simple way to improve a conventional A/O-MBR for high simultaneous carbon and nutrient removal from synthetic municipal wastewater
Source: PLoS One. 2019 Nov 22;14(11):e0214976. doi: 10.1371/journal.pone.0214976 (PMC6913871; doi:10.1371/journal.pone.0214976)
Supplement: S2 Table — (DOCX) [file pone.0214976.s002.docx]

**2S Table.** Oligonucleotide primers used for amplification

| **Primer** | **Sequence (5'-3')** | **Specificity** | **References** |
| --- | --- | --- | --- |
| amoA-1F | GGG GTT TCT ACT GGT GGT | AOB amoA gene | Rotthauwe et al. (1997) |
| amoA-2R | CCC CTC KGS AAA GCC TTC TTC | AOB amoA gene |  |
| Eub518F | ATT ACC GCG GCT GCT GG | All bacteria | Muyzer et al. (1993) |
| Eub338R | ACT CCT ACG GGA GGC AGC AG | All bacteria | Amman et al. (1990) |
| Cd3af | GTS AAC GTS AAG GAR ACS GG | NirS | Throbäck et al. (2004) |
| R3cd | GAS TTC GGR TGS GTC TTG A | NirS |  |
