# Supplementary material for: A simple way to improve a conventional A/O-MBR for high simultaneous carbon and nutrient removal from synthetic municipal wastewater
Source: PLoS One. 2019 Nov 22;14(11):e0214976. doi: 10.1371/journal.pone.0214976 (PMC6913871; doi:10.1371/journal.pone.0214976)
Supplement: S3 Table — (DOCX) [file pone.0214976.s003.docx]

3S Table: Reagents mixture composition for qPCR Method: SYBR Green 1

| **Reagent** | **Concentration (uL)** |
| --- | --- |
| ddH2O | 6.8 |
| Premix (LightCycler 480 SYBR Green 1 Master 2X) | 10 |
| Forward Primer (10uM) | 0.6 |
| Reverse Primer (10uM) | 0.6 |
| Mixture volume | 18 |
| DNA template | 2 |
| Total volume | 20 |
