# Supplementary material for: A simple way to improve a conventional A/O-MBR for high simultaneous carbon and nutrient removal from synthetic municipal wastewater
Source: PLoS One. 2019 Nov 22;14(11):e0214976. doi: 10.1371/journal.pone.0214976 (PMC6913871; doi:10.1371/journal.pone.0214976)
Supplement: S4 Table — (DOCX) [file pone.0214976.s004.docx]

**4S Table:** Phosphorus mass balance analysis in BF-A/O-MBR and C-A/O-MBR system.

| **Parameters** | **Unit** | **BF-A/O-MBR** | **C-A/O-MBR** |
| --- | --- | --- | --- |
| TP feeding rate (TP_inf._) | g-P/day | 0.061 ± 0.003 | 0.062 ± 0.003 |
| Anoxic incoming rate (Tp_inf._ + TP_Qr_) | g-P/day | 2.13 ± 0.31 | 0.24 ± 0.02 |
| TP releasing rate (∆P_release_) | g-P/day | 1.31 ± 0.47 | 0.03 ± 0.02 |
| Percent TP releasing | % | 61.81 ± 11.8 | 13.69 ± 10.2 |
| MBR incoming rate | g-P/day | 3.44 ± 0.71 | 0.26 ± 0.03 |
| TP uptake rate (∆P_uptake_) | g-P/day | 2.41 ± 0.58 | 0.18 ± 0.03 |
| Percent TP uptake | % | 69.98 ± 4.0 | 67.90 ± 2.4 |
| TP contained in MLSS | g-P/day | 0.015 ± 0.001 | 0.010 ± 0.002 |
| TP contained by weight of MLSS | % | 0.79 ± 0.1 | 0.08 ± 0.002 |
| TP removal by sludge wasting | g-P/day | 0.00 | 0.00 |
| TP in permeate | g-P/day | 0.001 ± 0.001 | 0.048 ± 0.005 |
| Overall TP Accumulation in the reactor | g-P/day | 0.060 ± 0.003 | 0.014 ± 0.001 |
| Sponge biomass | mg/g | 1804 ± 151 | - |
| TP content by weight of sponge biomass | % | 43.64 ± 10.6 | - |
| *(i(TP)_sponge_ )* |  |  |  |
| TP accumulated in sponge biomass | g-P/day | 0.044 ± 0.005 | - |
