# Supplementary material for: A simple way to improve a conventional A/O-MBR for high simultaneous carbon and nutrient removal from synthetic municipal wastewater
Source: PLoS One. 2019 Nov 22;14(11):e0214976. doi: 10.1371/journal.pone.0214976 (PMC6913871; doi:10.1371/journal.pone.0214976)
Supplement: S5 Table — (DOCX) [file pone.0214976.s005.docx]

**5S Table:** Relative abundance of bacterial at phyla level

| Phylum | BF-A/O-MBR (SP) | BF-A/O-MBR (SS) | C-A/O-MBR |
| --- | --- | --- | --- |
| Acidobacteria | 1.491 | 0.237 | 0.298 |
| Actinobacteria | 5.846 | 6.604 | 2.625 |
| Bacteroidetes | 13.227 | 18.063 | 14.169 |
| Chlamydiae | 0.022 | 0.398 | 0.195 |
| Chlorobi | 0.497 | 0.222 | 0.520 |
| Chloroflexi | 1.876 | 2.294 | 3.483 |
| Euryarchaeota | 1.076 | 0.002 | 0.020 |
| Firmicutes | 19.355 | 3.437 | 7.721 |
| Gemmatimonadetes | 0.017 | 0.086 | 0.359 |
| Minor phyla | 1.137 | 1.874 | 2.996 |
| Nitrospirae | 0.060 | 2.509 | 2.971 |
| Planctomycetes | 0.128 | 3.422 | 2.295 |
| Proteobacteria | 51.942 | 53.164 | 49.584 |
| Synergistetes | 0.023 | 0.007 | 0.014 |
| Tenericutes | 0.096 | 1.432 | 0.190 |
| Unclassified | 3.069 | 5.283 | 11.789 |
| Verrucomicrobia | 0.138 | 0.967 | 0.770 |
