# Supplementary material for: A simple way to improve a conventional A/O-MBR for high simultaneous carbon and nutrient removal from synthetic municipal wastewater
Source: PLoS One. 2019 Nov 22;14(11):e0214976. doi: 10.1371/journal.pone.0214976 (PMC6913871; doi:10.1371/journal.pone.0214976)
Supplement: S6 Table — (DOCX) [file pone.0214976.s006.docx]

**6S Table:** Relative abundance of bacterial at class level

| Class | BF-A/O-MBR (SP) | BF-A/O-MBR (SS) | C-A/O-MBR |
| --- | --- | --- | --- |
| Acidimicrobiia | 0.1827 | 0.2581 | 0.4353 |
| Acidobacteria | 0.0031 | 0.0149 | 0.0399 |
| Actinobacteria | 2.4747 | 6.0092 | 3.8410 |
| Alphaproteobacteria | 8.3164 | 15.8625 | 17.1085 |
| Anaerolineae | 1.8426 | 1.6889 | 1.8801 |
| Armatimonadia | 0.0001 | 0.0096 | 0.1397 |
| Bacilli | 9.7369 | 0.8884 | 0.8419 |
| Bacteroidia | 0.8159 | 0.0079 | 0.0136 |
| Betaproteobacteria | 33.4533 | 20.0816 | 19.5888 |
| Brachyspirae | 0.0035 | 0.0229 | 0.0052 |
| Brocadiae | 0.0099 | 0.3915 | 0.5194 |
| Caldisericia | 0.0009 | 0.0002 | 0.0004 |
| Caldithrixae | 0.0121 | 0.2051 | 0.2377 |
| Chlamydiia | 0.0224 | 0.3978 | 0.1954 |
| Chlorobia | 3.0450 | 0.1330 | 0.1528 |
| Chloroflexi | 0.0059 | 0.0044 | 0.0025 |
| Chrysiogenetes | 0.0001 | 0.0011 | 0.0005 |
| Chthonomonadetes | 0.0007 | 0.0190 | 0.0430 |
| Clostridia | 9.1313 | 2.3734 | 2.4362 |
| Deferribacteres | 0.0028 | 0.0017 | 0.0032 |
| Dehalococcoidetes | 0.0003 | 0.0075 | 0.0085 |
| Deinococci | 0.7265 | 0.0089 | 0.0060 |
| Deltaproteobacteria | 8.6486 | 5.2809 | 7.6415 |
| Elusimicrobia | 0.0001 | - | - |
| Epsilonproteobacteria | 0.0635 | 0.0948 | 0.1733 |
| Erysipelotrichi | 0.0003 | 0.0004 | 0.0024 |
| Fibrobacteria | 0.0012 | - | - |
| Fimbriimonadetes | 0.0020 | 0.2952 | 0.4114 |
| Flavobacteriia | 1.2894 | 0.2741 | 0.4280 |
| Fusobacteria | 0.0148 | 0.0018 | 0.0011 |
| Gammaproteobacteria | 11.1078 | 9.3622 | 13.2896 |
| Gemmatimonadetes | 0.0166 | 0.0859 | 0.3589 |
| Group II | 0.0362 | 0.0056 | 0.0041 |
| Holophagae | 0.1666 | 0.0191 | 0.0536 |
| Ignavibacteria | 0.4520 | 0.0890 | 0.3677 |
| Ktedonobacteria | 0.0207 | 0.2286 | 0.2037 |
| Leptospirae | 0.0905 | 0.0041 | 0.0064 |
| Methanobacteria | 0.0053 | 0.0005 |  |
| Methanomicrobia | 1.0705 | 0.0014 | 0.0203 |
| Methylacidiphilae | 0.0112 | 0.3715 | 0.2355 |
| Mollicutes | 0.0963 | 1.4319 | 0.1902 |
| Nitriliruptoria | 0.0025 | 0.0268 | 0.0216 |
| Nitrospira | 0.0597 | 2.5087 | 2.9705 |
| Nostocophycideae | 0.0360 | 0.0717 | 0.2163 |
| Opitutae | 0.1166 | 0.1921 | 0.2674 |
| Oscillatoriophycideae | 0.0628 | 0.0099 | 0.0118 |
| Pedosphaerae | 0.0053 | 0.0238 | 0.0353 |
| Planctomycetia | 0.1181 | 3.0180 | 1.7710 |
| Rubrobacteria | 0.0001 | 0.0001 | - |
| Solibacteres | 1.3206 | 0.2029 | 0.2045 |
| Spartobacteria | 0.0037 | 0.0271 | 0.1216 |
| Sphingobacteriia | 1.1088 | 17.7077 | 13.6988 |
| Spirochaetes | 0.1168 | 0.1038 | 0.1583 |
| Synechococcophycideae | 0.0022 | 0.0255 | 0.4372 |
| Synergistia | 0.0230 | 0.0072 | 0.0139 |
| Thermobacula | 0.0024 | 0.0054 | 0.0063 |
| Thermococci | 0.0001 | - | - |
| Thermodesulfobacteria | 0.0017 | 0.0017 | 0.0019 |
| Thermoleophilia | 0.1736 | 0.1781 | 0.1541 |
| Thermoprotei |  | 0.0001 | 0.0001 |
| Thermotogae | 0.0227 | 0.5761 | 0.2006 |
| Unclassified | 3.9422 | 9.0322 | 8.7296 |
| Verrucomicrobiae | 0.0007 | 0.3466 | 0.0919 |
| Zetaproteobacteria | - | - | 0.0001 |
