# Supplementary material for: A simple way to improve a conventional A/O-MBR for high simultaneous carbon and nutrient removal from synthetic municipal wastewater
Source: PLoS One. 2019 Nov 22;14(11):e0214976. doi: 10.1371/journal.pone.0214976 (PMC6913871; doi:10.1371/journal.pone.0214976)
Supplement: S7 Table — (DOCX) [file pone.0214976.s007.docx]

**7S Table:** Relative abundance of bacterial at genus level

| Genus | BF-A/O-MBR (SP) | BF-A/O-MBR (SS) | C-A/O-MBR |
| --- | --- | --- | --- |
| Agrobacterium | 2.3350 | 0.0849 | 0.0504 |
| Alicycliphilus | 2.9067 | 0.0837 | 0.0236 |
| Alkaliphilus | 2.4499 | 0.0622 | 0.0576 |
| Aminiphilus | 0.0116 | 0.0012 | 0.0090 |
| Amycolatopsis | 0.0002 | 0.3793 | 1.2456 |
| Azospira | 5.4278 | 2.3222 | 1.2012 |
| Azospirillum | 0.0389 | 1.9057 | 2.6957 |
| Candidatus Amoebophilus | 0.0054 | 6.8388 | 4.5694 |
| Clostridium | 0.6222 | 0.0676 | 0.0517 |
| Desulfobulbus | 2.6075 | 0.0026 | 0.0004 |
| Desulfovibrio | 0.0607 | 2.0902 | 2.4897 |
| Dyella | 0.3607 | 1.6405 | 2.1949 |
| Geobacillus | 5.3323 | 0.1295 | 0.1908 |
| Georgenia | 0.0012 | 0.3285 | 0.1530 |
| Hyphomicrobium | 0.4877 | 1.1851 | 0.9412 |
| Lewinella | 0.0013 | 1.9431 | 1.3190 |
| Luteimonas | 0.0081 | 0.0281 | 0.0402 |
| Methanosaeta | 0.0000 | 0.0002 | 0.0132 |
| Minor genus | 51.0394 | 53.3416 | 55.7889 |
| Mycobacterium | 0.0528 | 0.0926 | 0.0667 |
| Niastella | 0.0242 | 1.5015 | 0.7669 |
| Nitrospira | 0.0278 | 2.4076 | 2.8009 |
| Paucibacter | 0.1783 | 0.2629 | 0.2405 |
| Pelodictyon | 2.5843 | - | - |
| Phyllobacterium | 0.0286 | 3.5988 | 4.6518 |
| Propionispora | 4.1255 | 0.0044 | 0.0022 |
| Pseudomonas | 6.5958 | 0.2217 | 0.6531 |
| Runella | 0.0004 | 3.5758 | 2.7201 |
| Sphingobacterium | 0.0166 | 0.0438 | 0.0439 |
| Steroidobacter | 0.2830 | 1.4841 | 1.2920 |
| Sutterella | 0.0015 | 0.0004 | 0.0015 |
| Thauera | 11.3973 | 0.0815 | 0.0822 |
| Thiocapsa | 0.0078 | 1.5546 | 3.7624 |
| Thiomonas | 0.0204 | 2.8395 | 3.1115 |
| Trichococcus | 0.0003 | 0.0031 | 0.0019 |
| Verrucomicrobium | - | 0.2804 | 0.0727 |
| Vogesella | 0.1254 | 0.7470 | 0.6669 |
| Zoogloea | 0.8337 | 8.8654 | 6.0273 |
