# Supplementary figures and images for: A simple way to improve a conventional A/O-MBR for high simultaneous carbon and nutrient removal from synthetic municipal wastewater
Source: PLoS One. 2019 Nov 22;14(11):e0214976. doi: 10.1371/journal.pone.0214976 (PMC6913871; doi:10.1371/journal.pone.0214976)

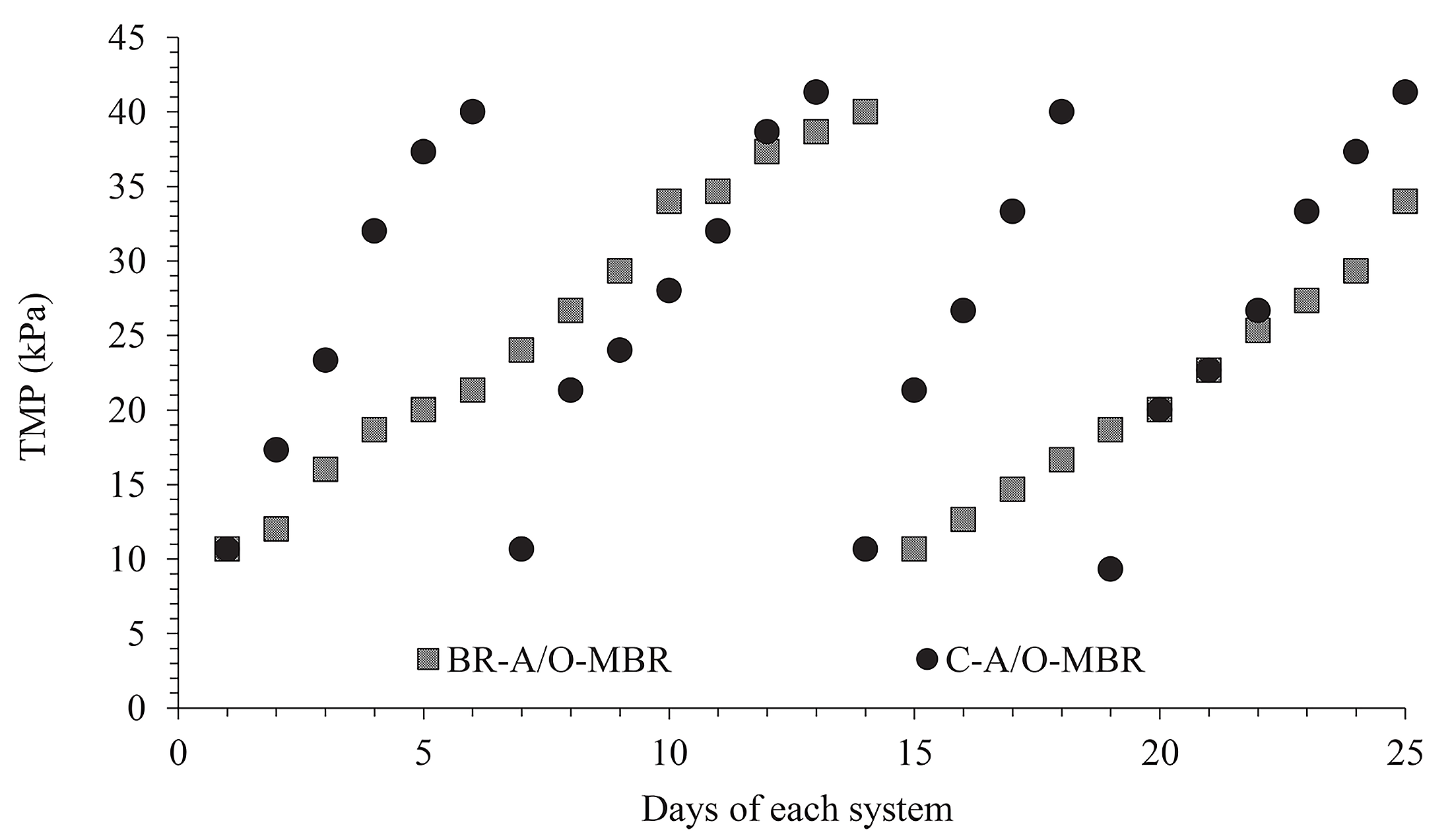

Supplement: S1 Fig — (TIF) [file pone.0214976.s008.tif]
